# Test accuracy of artificial intelligence-based grading of fundus images in diabetic retinopathy screening: A systematic review

**DOI:** 10.1177/09691413221144382

**Published:** 2023-01-09

**Authors:** Zhivko Zhelev, Jaime Peters, Morwenna Rogers, Michael Allen, Goda Kijauskaite, Farah Seedat, Elizabeth Wilkinson, Christopher Hyde

**Affiliations:** 1 Exeter Test Group, University of Exeter Medical School, 3286 University of Exeter, Exeter, UK; 2 NIHR ARC South West Peninsula (PenARC), 171002 University of Exeter Medical School, University of Exeter, Exeter, UK; 3 University of Exeter Medical School, 3286 University of Exeter, Exeter, UK; 4 UK National Screening Committee, London, UK; 5 9553 Northern Devon Healthcare NHS Trust, Devon, UK

**Keywords:** Artificial intelligence, fundus imaging, sensitivity and specificity, systematic review, diabetic retinopathy, screening

## Abstract

**Objectives:**

To systematically review the accuracy of artificial intelligence (AI)-based systems for grading of fundus images in diabetic retinopathy (DR) screening.

**Methods:**

We searched MEDLINE, EMBASE, the Cochrane Library and the ClinicalTrials.gov from 1st January 2000 to 27th August 2021. Accuracy studies published in English were included if they met the pre-specified inclusion criteria. Selection of studies for inclusion, data extraction and quality assessment were conducted by one author with a second reviewer independently screening and checking 20% of titles. Results were analysed narratively.

**Results:**

Forty-three studies evaluating 15 deep learning (DL) and 4 machine learning (ML) systems were included. Nine systems were evaluated in a single study each. Most studies were judged to be at high or unclear risk of bias in at least one QUADAS-2 domain. Sensitivity for referable DR and higher grades was ≥85% while specificity varied and was <80% for all ML systems and in 6/31 studies evaluating DL systems. Studies reported high accuracy for detection of ungradable images, but the latter were analysed and reported inconsistently. Seven studies reported that AI was more sensitive but less specific than human graders.

**Conclusions:**

AI-based systems are more sensitive than human graders and could be safe to use in clinical practice but have variable specificity. However, for many systems evidence is limited, at high risk of bias and may not generalise across settings. Therefore, pre-implementation assessment in the target clinical pathway is essential to obtain reliable and applicable accuracy estimates.

## Background

Diabetic retinopathy (DR) is one of the most common complications of diabetes. Screening for DR aims to identify and monitor patients with more advanced forms of DR, so that treatment can be administered when it is most effective.^1^ Furthermore, screening might have indirect benefits, by increasing patients’ awareness and motivation, leading to better management of diabetes and slower progression of DR.^2–4^ Diabetic eye screening programmes (DESP) are cost-effective relative to no screening^5^ and data from epidemiological studies suggest that they are effective in reducing progression to proliferative DR (PDR) and preventing visual loss.^6–8^ However, they are costly to run and require a highly trained workforce and accessible diabetes and eye care services. Even well-established programmes, such as those in the UK, face challenges due to the increasing number of patients with diabetes.^9^

In recent years, attempts to improve the efficiency of DESPs focused mainly on risk-stratified screening^9,10^ and the introduction of artificial intelligence (AI)-based automated retinal imaging assessment systems (ARIASs). In 2011, the first ARIASs were introduced in the Scottish and Portuguese DESPs to rule out DR prior to human grading. Given the large proportion of patients with normal images^11^ and the low risk of the software missing clinically significant DR, both implementations were considered successful.^12,13^

However, despite the initial success and the increasing interest that followed the advent of deep learning (DL) algorithms, the introduction of ARIASs in clinical practice has been slower than expected and there are still concerns about the safety, cost-effectiveness and overall impact of AI-based screening. The current paper provides an up-to-date review of the accuracy and safety of ARIASs that are in the final stages of their clinical evaluation. It is based on the results from a larger project commissioned by the UK National Screening Committee (NSC) which, among its other components, included a systematic review of the accuracy of ARIASs. The review protocol was registered on PROSPERO (CRD42020200515) and any amendments are detailed and justified below.

## Methods

We developed a search strategy combining free text and medical subject headings for ‘diabetic retinopathy’, ‘screening’ and ‘ARIAS’ (Supplemental material, Table S1) and searched MEDLINE (via OvidSp), EMBASE (via OvidSp), the Cochrane Library (CDSR and CENTRAL) and ClinicalTrials.gov (U.S. National Library of Medicine) from 1st January 2000 to 27th August 2021. In addition, we searched the reference lists of all included studies and relevant papers and contacted experts to check for additional titles.

English-language papers were included if they reported an evaluation of ARIAS in an external dataset (i.e. different from the one used for development); participants were ≥12 years of age; had type 1 or 2 diabetes; and underwent standard fundus photography to detect DR. The review was commissioned to inform a discussion on the use of ARIASs in the UK and, therefore, the original analysis published in the project's report^14^ focused on studies applicable to the UK DESPs. The analysis presented here is not restricted to UK-relevant studies and has a slightly different focus: on the accuracy of ARIASs that are commercially available, licensed for clinical use or evaluated in a ‘clinically plausible dataset’ (defined as a dataset obtained from a well-characterised cohort of patients from the target population). When an evaluation in multiple datasets was reported, we included only the results from the most clinically relevant cohort. The other differences between the original and current analysis are as follows: (1) we excluded studies in which the ARIAS was used only as a decision aid in manual grading; (2) we excluded ARIASs evaluated only in publicly available datasets or poorly characterised datasets as those are more characteristic of the earlier stages of the evaluation process; (3) we excluded studies reporting accuracy at lesion level only or when no sensitivity and specificity estimates were reported or could be calculated from the published data; (4) we excluded conference abstracts or similar publications as those do not report study methods and results in sufficient detail.

Search results were imported into EndNote X8.2 (Thomas Reuters). A single reviewer carried out the screening of titles/abstracts and full text, with a second reviewer screening independently 20% of the titles at each level and resolving disagreements through discussion. This process was repeated for data extraction and methodological quality appraisal. The latter was carried out using the QUADAS-2 tool^15^ and QUADAS-2C extension for comparative studies.^16^ While in the original analysis applicability was assessed against the UK DESP, here we adopted broader criteria that reflect the variation across screening programmes (Tables S2 and S3). Given the small number of studies evaluating each ARIAS and the considerable clinical heterogeneity and high risk of bias, we summarised the results in tables and by plotting them in the Receiver Operating Characteristics (ROC) space and analysed them narratively. When not reported in the study, confidence intervals were recalculated from the accuracy estimates, prevalence and sample size. Review Manager 5.4.1 (The Cochrane Collaboration, 2020) was used to create the ROC plot and to calculate the confidence intervals around the sensitivity and specificity estimates.

## Results

Our database searches identified 3309 records. Of those 854 were duplicates; 2455 were screened at title/abstract level and 387 were assessed at full text. The agreement (Cohen's kappa) between the reviewers who conducted the screening was 0.79 (titles/abstracts) and 0.83 (full text). Ultimately, 43 papers were included in the current analysis (Figure S1). Studies excluded at full text, with reasons for exclusion, are provided in Table S4.

### Study characteristics

The studies evaluated 15 DL-based and 4 traditional machine learning (ML)-based systems^11,17–27^; both DL and ML versions of EyeArt were included. Two studies reported head-to-head comparison of more than one system.^22,27^ Nine systems were evaluated in a single study each; and only six were evaluated in ≥3 studies (Table S5). The populations from which the external datasets were drawn were from the following countries: Australia, Chile, China, Denmark, France, India, Italy, New Zealand, Poland, Portugal, Singapore, Spain, Thailand, the Netherlands, UK, USA and Zambia. Ten studies recruited participants prospectively, with another three^28–30^ implying, but not explicitly claiming, a prospective design. Seven studies compared head-to-head the accuracy of the system to that of human graders not involved in the reference grading.^25,31–36^ Only five studies were conducted independently from the developer/manufacturer^22,27,29,37,38^; and another one appeared to be an independent evaluation but without stating this explicitly^26^ (Table 1).

**Table 1.** Characteristics of the included studies.

Study	Origin of the external dataset^a^	Prospective	Independent^b^	Compared to human graders	Setting	Photographic protocol	Reference standard
*Airdoc (DL)*							
He 2020^28^	China	Probably	No	No	Community hospital	45°, 2 fields, no mydriasis	Two ophthalmologists, independently
*DAPHNE (DL)*							
Al Turk 2020^39^	China	No	No	No	DR screening	45°, 2 fields, mydriasis not reported	Trained and certified graders (no further details)
*DART (DL)*							
Arenas-Cavalli 2021^40^	Chile	No	No	No	PC	45°, 2 fields, no mydriasis	A single ophthalmologist (out of 8)
*DeepDR (DL)* ^c^							
Dai 2021^41^	China	No	No	No	DM study cohort	45°, 2 fields, no mydriasis	Images read independently by two certified ophthalmologists (reading group of 10 experts, ≥7 years of experience) with disagreements adjudicated by a senior supervisor; 20% of the grading results were randomly re-read to check for consistency
*DLA (DL)*							
Baget-Bernaldiz 2021^42^	Spain	No	No	No	SpDESP	45°, 1 field, no mydriasis	Four retinal specialists masked to DLA results independently read images; disagreement resolved by discussion and consensus
Romero-Aroca 2020^43^	Spain	No	No	No	SpDESP	45°, 1 field, no mydriasis	Four retinal specialists masked to DLA results read images; unclear if this was done independently for each image, as method of adjudication not reported
*EyeArt v1 (ML) and v2 (DL)*							
Bhaskaranand 2016 (ML)^17^	USA (other)	No	No	No	EyePACS	N/A	A human expert
Bhaskaranand 2019 (DL)^44^	USA	No	No	No	EyePACS	45°, 3 fields; mydriasis in 45.8% of participants	EyePACS trained and certified optometrists and ophthalmologists; a subset of 192 randomly selected patient encounters re-graded by an expert at the Doheny Eye Institute
FDA 2020 (DL)^45^	USA	Yes	No	No	PC and OPH	45°, 2 fields; mydriasis if ungradable	FPRC using 4W-D protocol and independent grading by 2 certified graders, adjudication
Heydon 2020 (DL)^37^	UK	Yes	Yes	No	EDESP	45°, 2 fields; mydriasis	The final grade from the EDESP manual grading
Liu 2020 (DL)^46^	USA	Yes	No	No	PC	Not reported; no mydriasis	A single retina specialist (1 out of 5 specialists involved in the study)
Olvera-Barrios 2020 (DL)^38^	UK	No	Yes	No	EDESP	45°, 2 fields, mydriasis; standard fundus photography compared to EIDON confocal scanner (CenterVue, Padua, Italy)	The final grade from the EDESP manual grading
Sarao 2020 (DL)^29^	Italy	Unclear	Yes	No	Routine DM visit	45°, 1 field, no mydriasis; standard fundus photography compared to EIDON confocal scanner (CenterVue, Padua, Italy)	2 retinal specialists, masked to each other and any patient records; adjudication by a third specialist
*EyeGrader (DL)*							
Keel 2018^47^	Australia	Yes	No	No	Hospital	45°, 1 field, no mydriasis	An ophthalmologist
Li 2018^48^	Australia, Singapore	No	No	No	NIEHS, SiMES, AusDiab	45°, 2 fields, use of mydriasis varied across cohorts	Trained professional graders
*EyeWisdom (DL)*							
Ming 2021^49^	China	Yes	Unclear	No	PC	45°, 1 field, no mydriasis	Two licensed ophthalmologists, independently; masked to the AI results; adjudication by a panel, majority vote
*Google AI (DL)*							
Gulshan 2019^31^	India	Yes	No	Yes	2 tertiary eye care centres	40°–45°, 1 field, no mydriasis	Site 1: 3 retinal specialists, independently, final grade by consensus; Site 2: only disagreements were adjudicated by the above panel
Gulshan 2016^50^	USA	No	No	No	EyePACS-1	45°, 1 field, mydriasis in 40% of participants	Graded by 8 US board certified ophthalmologists independently, final grade: simple majority decision
Krause 2018^32^	USA	No	No	Yes	EyePACS-2	45°, 1 field, mydriasis not reported	3 retinal specialists, adjudication by consensus
Raumviboonsuk 2019^33^	Thailand	No	No	Yes	Diabetes registry	45°, 1 field, mydriasis not reported	Retina specialists, who graded all disagreements and 5% of the images where the algorithm and regional graders agreed
*IDx-DR*							
Abramoff 2016^51^	France	No	No	No	Messidor-2	45°, 1 field, mydriasis at 2 of 3 sites	3 board certified retinal specialists independently graded all images, adjudication by consensus
Abramoff 2018^52^	USA	Yes	No	No	PC	45°, 2 fields, mydriasis in 23.6% of participants	FPRC using 4W-D protocol and independent grading with adjudication; OCT for DMO
Shah 2020^53^	Spain	No	No	No	SDESP	45°, 2 fields, mydriasis	3 ophthalmologists, exams lacking consensus were adjudicated by a retinal specialist
van der Heijden 2018^54^	Netherlands	Yes	No	No	PC	45°, 2 fields, no routine mydriasis	3 retinal specialists, independently, adjudication by consensus
Verbraak 2019^55^	Netherlands	No	No	No	PC	45°, 2 fields, mydriasis if needed	2 readers, independently, adjudicated by a retinal specialist
*RedCAD*							
Gonzalez-Gonzalo 2020^34^	France	No	No	Yes	Messidor	45°, 1 field; mydriasis in 66.7% of participants	Messidor (medical experts, no further details)
*SELENA (DL)*							
Bellemo 2019^56^	Zambia	Yes	No	No	DESP	45°, 2 field, mydriasis not reported	Two professional senior graders (does not state independent grading and no adjudication reported)
Ting 2017^35^	Singapore, other	No	No	Yes	SiDRP 2014–2015; 10 multi-ethnic cohorts	SIDRP: 45°, 2 fields, mydriasis not reported; other cohorts: not reported	SiDRP: single retinal specialist with >5 years of experience; multi-ethnic cohorts – various reference standards
*Visiona (DL)*							
Ramachandran 2018^57^	New Zealand	No	Unclear	No	DESP	45°, at least 2 posterior pole images (a macula centred and a macula off-centred temporally by one disc diameter) and 1 nasal image, mydriasis in 75% of participants	An accredited ophthalmic medical photographer using the NZ MoH guideline, followed by an ophthalmologist if grading by a secondary grader was required (10.3% of eyes graded by the primary grader only)
*Other DL systems*							
Cen 2021^36^	China	No	No	Yes	Hospital	Not reported for external dataset (primary dataset: 35°–50°, 1 field, mydriasis not reported)	Images labelled by an unspecialised ophthalmologist with >3 years training and then confirmed by a senior retina specialist with >7 years of experience (10 such pairs were involved); disagreements were adjudicated by a retina expert panel of 5 senior retinal specialists
Kanagasingam 2018^30^	Australia	Probably	No	No	PC	45°, 1 field, 1–3 images per eye allowed, mydriasis not reported	A single ophthalmologist
*iGradingM (ML)*							
Fleming 2010a^11^	UK, Scotland	No	No	No	SDESP	45°, 1 field, mydriasis if needed	The SDESP's final grade; arbitration by 7 senior ophthalmologists on referrals by the SDESP that were missed by the software
Fleming 2010b^20^	UK, Scotland	No	No	No	SDESP	Not reported (probably as per the SDESP protocol)	The SDESP's manual grading + a research fellow's grading, arbitrated by a lead clinician
Goatman 2011^21^	UK, England	No	No	No	EDESP	45°, 2 fields (1 and 2 fields compared), mydriasis	The EDESP's final grade + 2 levels of arbitration of disagreements between the system and EDESP grade
Philip 2007^25^	UK, Scotland	Yes	No	Yes	SDESP	45°, at least 1 disc/macula photograph per screenable eye, mydriasis if needed (21.2% of patients)	A single grader (trained clinical research fellow)
Soto-Pedre 2015^26^	Spain	No	Probably	No	SpDESP	45°, 1 field, mydriasis if needed	The SpDESP's final grade
*RetinaLyze (ML)*							
Bouhaimed 2008^18^	UK, Wales	No	No	No	WDESP	45°, 2 fields, mydriasis	The WDESP's final grade (each image was assessed by a team of senior clinician, diabetologist, and ophthalmologist)
Hansen 2004^23^	Denmark	No	No	No	hospital	45°, 5 overlapping non-stereoscopic photographs of each eye, without and with mydriasis	2 independent graders, disagreements adjudicated by a third grader
*RetmarkerSR (ML)*							
Figueiredo 2015^19^	Portugal	No	No	No	PDESP	45°, 2 fields, no mydriasis (in 2 of the 4 datasets a few patients had less than 4 images per patient)	The PDESP's final grade
Oliveira 2011^24^	Portugal	No	No	No	PDESP	45°, 2 fields, no mydriasis	PDESP reading centre, all images graded by an experienced ophthalmologist
Ribeiro 2015^13^	Portugal	No	No	No	PDESP	45°, 2 fields, no mydriasis	The PDESP's final grade
*Studies comparing >1 system*							
Tufail 2016^27^	UK, England	No	Yes	No	EDESP	45°, 2 fields, mydriasis	The EDESP's final grade; pre-specified subsets of results were sent for arbitration at a US-based fundus photographic reading centre
Grzybowski 2021^22^	Poland	No	Yes	No	Hospital	45°, 2 fields, no mydriasis	A single ophthalmologist with basic experience with DR screening

AFEDS: African American Eye Disease Study; DESP: Diabetic Eye Screening Programme; DL: deep learning; EDESP: English DESP; ML: machine learning; OPH: ophthalmology; PC: primary care; PDESP: Portuguese DESP; RDR: referable diabetic retinopathy; RS: reference standard; SDESP: Scottish DESP; SiDRP: Singapore DESP; SpDESP: Spanish DESP; WDESP: Welsh DESP.

^a^ These are the external datasets for which accuracy estimates were included in the review; datasets used for training, internal validation and some of the less relevant external datasets (e.g. public datasets) are not included.

^b^ By ‘independent’ we mean that the developer/manufacturer of the system was not involved in or provided funding for the study.

^c^ We found another study evaluating DeepDR, Wang 2020,^58^ but the paper was poorly written and difficult to understand; the external cohorts were poorly characterised and it was unclear if the external datasets overlapped with those reported in Dai 2021; ultimately, we decided that the study should be excluded.

The sample size ranged from 83^23^ to 107,001 participants.^44^ Most of the studies included patients enrolled in DESPs or outpatient diabetes clinics. However, the inclusion criteria and patient characteristics varied considerably and were reported inconsistently. In particular, studies varied on patient age as an eligibility criterion (range ≥12 to >40 years of age), and with respect to the distribution of age, race/ethnicity, duration of diabetes and HbA1c level in the included patients. The prevalence of referable diabetic retinopathy (RDR) ranged from 1%^30^ to 47%^29^ suggesting considerable variation in the distribution of DR grades across study samples.

### Methodological quality

The methodological quality of the included studies is summarised in Table 2. Only 11/43 studies were judged to be at low risk of bias in the patient selection domain and 29/43 and 26/43 in the reference standard and flow & timing domains, respectively. In contrast, all but four studies were found to be at low risk of bias in the index test domain. The main issues were failure to include a representative sample of the target population at patient and/or image level (e.g. convenience sampling, not reporting the method of sampling in sufficient detail or excluding images of low quality); failure to meet the reference standard criterion of at least two independent experts grading each image; and exclusion of technical failures from the analysis. Most of the studies were judged to be at low risk of applicability concerns across all three domains.

**Table 2.** Methodological quality of included studies using the QUADAS-2 checklist.

ARIAS	Study	PS: RB	PS: A	IT: RB	IT: A	RS: RB	RS: A	F&T: RB
Airdoc (DL)	He 2020	Unclear	Low	Low	Low	Low	Low	High
Cen 2021 (DL)	Cen 2021	Unclear	Unclear	Low	Unclear	Low	Low	High
DAPHNE (DL)	Al Turk 2020	Unclear	Unclear	Low	Low	Unclear	Low	Unclear
DART (DL)	Arenas-Cavalli 2021	High	Low	Low	Low	High	Low	Low
DeepDR (DL)	Dai 2021	Unclear	Unclear	Low	Low	Low	Low	Unclear
DLA (DL)	Baget-Bernaldiz 2021	Low	High	Low	Low	Low	Low	High
DLA (DL)	Romero-Aroca 2020	Low	Low	Low	Low	Unclear	Low	High
EyeArt v1 (ML)	Bhaskaranand 2016	Unclear	Low	Low	Unclear	High	Low	Low
EyeArt v2 (DL)	Bhaskaranand 2019	High	Low	Low	Low	Low	Low	Low
EyeArt v2 (DL)	Heydon 2020	Low	Low	Low	Low	Low	Low	Low
EyeArt v2 (DL)	FDA 2020	High	Low	Low	Low	Low	Low	Low
EyeArt v2 (DL)	Liu 2020	Unclear	Low	Low	Low	High	Low	Low
EyeArt v2 (DL)	Olvera-Barrios 2020	High	Low	Low	Low	Low	Low	Low
EyeArt v2 (DL)	Sarao 2020	Unclear	Low	Low	Low	Low	Low	High
EyeGrader (DL)	Keel 2018	High	Low	Low	Low	Unclear	Unclear	Unclear
EyeGrader (DL)	Li 2018	High	High	Low	Low	High	Low	Low
EyeWisdom (DL)	Ming 2021	Unclear	Low	Low	Low	Low	High	Low
Google (DL)	Gulshan 2016	Unclear	Low	Low	Low	Low	Low	Low
Google (DL)	Gulshan 2019	High	High	Low	Low	Low	Low	Low
Google (DL)	Krause 2018	Unclear	Low	Low	Low	Low	Low	Unclear
Google (DL)	Raumviboonsuk 2019	Unclear	Low	Low	Low	Low	Low	High
IDx-DR (DL)	Abramoff 2016	High	Low	Low	Low	Low	Low	Low
IDx-DR (DL)	Abramoff 2018	High	Low	Low	Low	Low	Low	Low
IDx-DR (DL)	Shah 2020	High	Low	Low	Low	Low	Low	High
IDx-DR (DL)	van der Heijden 2018	High	Low	Low	Low	Low	Low	High
IDx-DR (DL)	Verbraak 2019	High	Low	Low	Low	Low	Low	High
iGradingM (ML)	Fleming 2010a	Low	Low	Low	Low	Low	Low	Low
iGradingM (ML)	Fleming 2010b	High	Low	Low	Low	Low	Low	Low
iGradingM (ML)	Goatman 2011	Low	Low	Low	Low	Low	Low	Low
iGradingM (ML)	Philip 2007	Low	Low	Low	Low	High	Low	Low
iGradingM (ML)	Soto-Pedre 2015	Low	Low	Low	Low	Low	Low	High
Kanagasingam 2018 (DL)	Kanagasingam 2018	Low	Low	Low	Low	High	Low	Low
RedCAD (DL)	Gonzalez-Gonzalo 2020	High	Low	Low	Low	Unclear	Low	Unclear
RetinaLyze (ML)	Bouhaimed 2008	Low	Low	Low	Low	Low	Low	Low
RetinaLyze (ML)	Hansen 2004	High	Low	High	Low	Low	High	Low
RetmarkerSR (ML)	Figueiredo 2015	High	Low	Unclear	Low	Low	Low	Low
RetmarkerSR (ML)	Oliveira 2011	High	Low	High	Low	Low	Low	Low
RetmarkerSR (ML)	Ribeiro 2015	Low	Low	Low	Low	Low	Low	Low
SELENA (DL)	Bellemo 2019	Unclear	Low	Low	Low	High	Low	High
SELENA (DL)	Ting 2017	Unclear	Low	Low	Low	High	Low	Low
Visiona (DL)	Ramachandran 2018	High	Low	High	Low	High	Low	High
EyeArt v1 (ML), RetmarkerSR (ML), iGradingM (ML)	Tufail 2016	Low	Low	Low	Low	Low	Low	Low
IDx-DR (DL), Retinalyze (ML)	Grzybowski 2021	High	Low	Low	Low	High	Low	Low

A: applicability; ARIAS: artificial intelligence-based automated retinal imaging assessment system; DL: deep learning; I: index test domain; ML: machine learning; PS: patient selection domain; RB: risk of bias; RS: reference standard domain; F&T: flow and timing domain.

### Test accuracy of ARIASs

**Figure 1.**
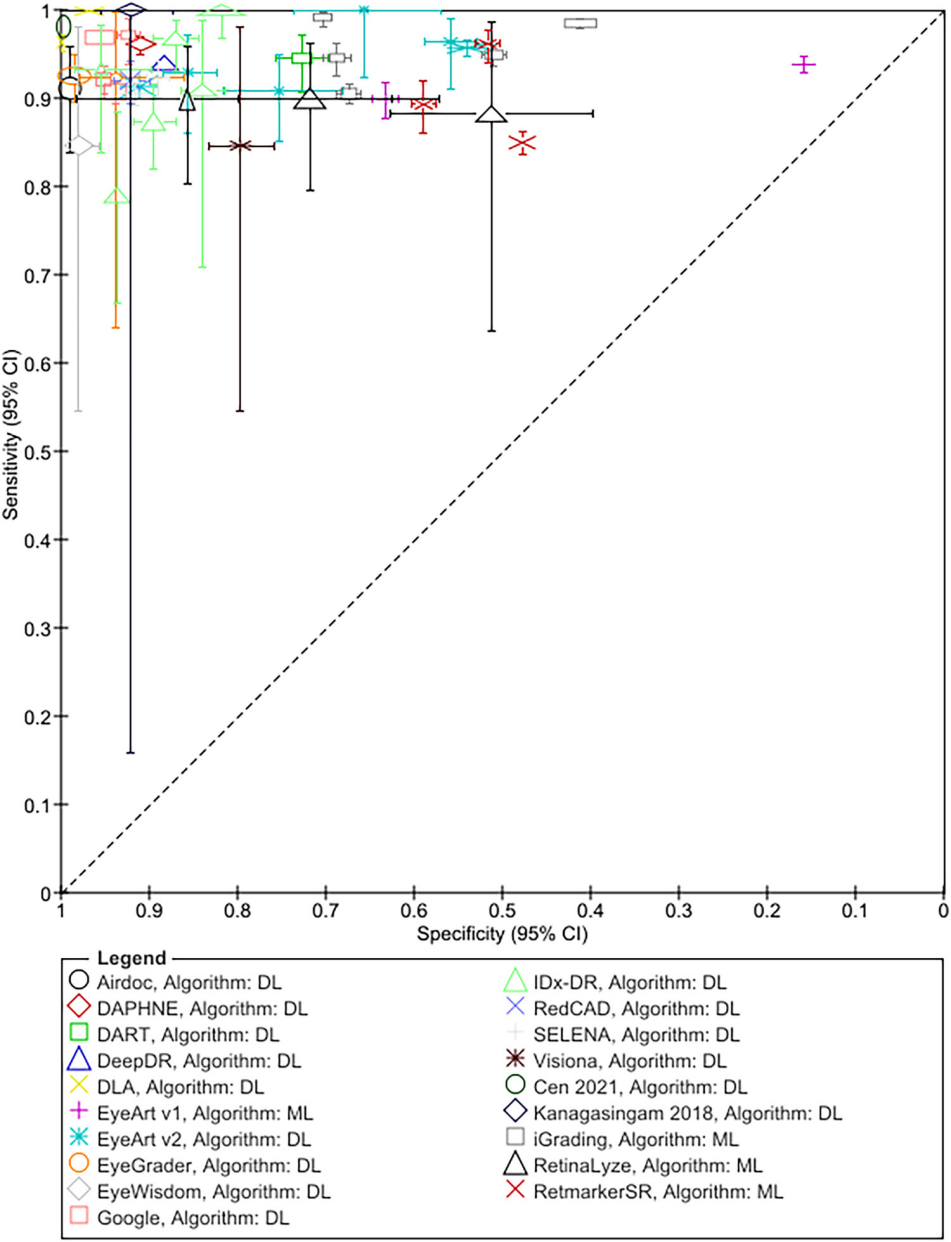
Summary ROC plot of study-level sensitivity and specificity by AI system (given the clinical and methodological variability across studies, the results should not be interpreted as an indication of the comparative accuracy of the systems).

The results for RDR are summarised in Figure 1 and Table 3; Tables S6 and S7 detail sensitivity, specificity, and positive and negative predictive values arranged by prevalence of DR in the study samples. In addition, Table S8 details accuracy at other thresholds and factors affecting accuracy. RDR was commonly defined as a moderate or worse non-proliferative DR (NPDR) or diabetic macular oedema (DMO). All but two studies^54,55^ reported sensitivities ≥85%. Specificity estimates, on the other hand, varied widely ranging from 20%^27^ to 100%.^42^ All 13 studies evaluating ML systems reported specificities <80%, compared to only 6 of the 31 studies evaluating DL systems (Figure 1, Table 3).

**Table 3.** Test accuracy for referable diabetic retinopathy.

Study	Sample size (*N*)	Prevalence of RDR (%)	Definition of RDR (grading criteria)	Sensitivity for RDR, % (95% CI)	Specificity for RDR, % (95% CI)	Characteristics of FNs
*Airdoc (DL)*						
He 2020	889 P	11.4	mtmDR or DMO (ICDR)	91.18 (86.4 to 94.7)	98.79 (98.1 to 99.3)	N/A
*DAPHNE (DL)*						
Al Turk 2020^a^	15,000 Im	11.6	R2, R3, DMO (EDESP)	95.51 (93.1 to 97.50)	91.11 (85.11 to 92.63)	PDR detection rate 100% (95.8% to 100%)
*DART (DL)*						
Arenas-Cavalli 2021	1123 P	19.4	R2, R3, R4, DMO, U (EURODIAB)	94.5 (91.0 to 97.0)^b^	72.7 (70.0 to 76.0)^b^	N/A
*DeepDR (DL)*						
Dai 2021^c^	23,186 P	N/A	Moderate NPDR or worse, DMO or both (ICDR)	94.0 (93.6 to 94.4)	88.3 (88.1 to 88.5)	N/A
*DLA (DL)*						
Baget-Bernaldiz 2021	7164 P	10.6	Moderate NPDR or worse or referable DMO (ICDR)	96.7 (95.69 to 99.49)	99.92 (99.85 to 99.96)	1) 9 images: no DR by DLA, but moderate DR by RS; 2) 4 images: no DR by DLA, but severe DR by RS; 3) 32 images: mild DR by DL, but moderate DR by RS; 4) 13 images: mild DR by DLA, but severe DR by RS
Romero-Aroca 2020	38,694 Im	10.0 Im	Moderate NPDR or worse (ICDR)	99.8 (99.0 to 100)^b^	96.8 (96.0 to 97.0)^b^	Not reported
*EyeArt (ML and DL)*						
Bhaskaranand 2016 (ML)	5084 V	16.9 V	mtmNPDR or MO (ICDR)	90.0 (88.0 to 92.0)	63.2 (61.7 to 64.6)	77/87 FNs did not meet the treatment criteria
Bhaskaranand 2019 (DL)	107,001 V	19.3	Moderate or severe NPDR, PDR, and/or CSDMO (ERGS)	91.3 (90.9 to 91.7)	91.1 (90.9 to 91.3)	95.4% of FNs did not meet general treatment criteria
FDA 2020 (DL)^d^	655 P	N/A	Moderate NPDR or higher (ICDR) or CSDMO	Range 92.9 to 100 across cohorts	Range 85.2 to 92.0 across cohorts	N/A
Heydon 2020 (DL)	30,405 V	7.2	U, M1, R2 and R3 (EDESP)	95.7 (94.8 to 96.5)	54.0 (53.4 to 54.5)	No R2, R3 or DMO were missed
Liu 2020 (DL)	180 P	25.6	Moderate or worse DR or DMO or inconclusive (ICDR)	100 (92.3 to 100)	65.7 (57.0 to 73.7)	All cases of VTDR were identified
Olvera-Barrios^e^ 2020 (DL)	1257 P	8.43	U, M1, R2 and R3 (EDESP)	97 (91 to 99)	55.9 (53.0 to 59.0)^b^	Identified all cases of VTDR
Sarao 2020 (DL)^e^	322 E	47.2 E	R1M1, R2M0, R2M1, R3M0, R3M1 (UK NHS)	90.8 (85.0–94.9)	75.3 (68.0–81.7)	N/A
Tufail 2016 (ML)	20,258 P	13.7	U, M1, R2 and R3 (EDESP)	93.8 (92.9 to 94.6)	20 (19 to 21)	Identified >99% of R2M1, R3M0 and R3M1
*EyeGrader (DL)*						
Keel 2018	96 P	13.5	Moderate NPDR or worse, or DMO (EDESP)	92.3 (64.0 to 100)^b^	93.7 (86.0 to 98.0)^b^	1 FN case of stable PDR with subthreshold panretinal laser scars
Li 2018^f^	13,657 E	2.9	Pre-proliferative DR or worse, or DMO (EDESP)	92.5 (89.0 to 95.0)^b^	98.5 (98.0 to 99.0)^b^	n/a
*EyeWisdom (DL)*						
Ming 2021^g^	173 P	10.7 E (any DR)	mtm NPDR (ICDR); DMO not reported	84.6 (54.6 to 98.1)	98.0 (94.3 to 99.6)	FNs: 4 moderate NPDR, 1 severe NPDR
*Google AI (DL)*						
Gulshan 2016	9963 Im	DR: 7.8, DMO: 3.1, Im	Moderate or worse DR or DMO or U (ICDR)	1) 90.7 (89.2 to 92.1) 2) 96.7 (95.7 to 97.5)	1) 93.8 (93.2 to 94.4) 2) 84.0 (83.1 to 85.0)	n/a
Gulshan 2019^h^	3049 P	>30	Moderate or worse DR or referable DMO (ICDR)	DR: Site 1: 88.9 (85.8 to 91.5) Site 2: 92.1 (90.1 to 93.8) DMO: Site 1: 97.4 (95.2 to 98.8) Site 2: 93.6 (91.3 to 95.4)	DR: Site 1: 92.2 (90.3 to 93.8) Site 2: 95.2 (94.2 to 96.1) DMO: Site 1: 90.7 (88.9 to 92.3) Site 2: 92.5 (91.3 to 93.5)	n/a (but 28 of 139 FNs when using the new model were grade 3 or 4 (ICDR), see footnote)
Krause 2018	998 P	11.6 Im	Moderate or worse DR or referable DMO (ICDR)	RDR: 97.1 (94.00 to 99.0)^b^ DMO: 94.9	RDR: 92.3 (91.0 to 94.0)^b^ DMO: 94.4	Missed 6 cases of moderate NPDR, but no severe or PDR; also missed 4 cases of referable DMO
Raumviboonsuk 2019	7517 P	12.17	Moderate NPDR or worse or DMO (ICDR)	RDR: 96.8 (96.0 to 97.0)^b^ (range: 89.3 to 99.3); DMO: 95. 3 (range: 85. 9 to 100)	RDR: 95.6 (95.0 to 96.0)^b^ (range: 98.3 to 98.7); DMO: 98.2 (range: 94.4 to 99.1)	Missed 3.3% of severe NPDR or worse images; and 4.5% of PDR images
*IDx-DR (DL)*						
Abramoff 2016	874 P	21.7	Moderate or worse DR or DMO (ICDR)	96.8 (93.3 to 98.8)	87.0 (84.2 to 89.4)	No cases of severe NPDR, PDR or DMO were missed
Abramoff 2018	819 P	23.8	mtmDR or DMO (ETDRS); RS: 1) 4W-D; 2) 4W-D & OCT	1) 87.2 (81.8 to 91.2) 2) 85.9 (82.5 to 88.7)	1) 90.7 (88.3 to 92.7) 2) 90.7 (86.8 to 93.5)	1) 37/38 cases and 2) 47/51 case of VTDR identified, respectively
Grzybowski 2021^i^	170 P	N/A	mtm DR (ICDR)	93.33 (83.80 to 98.15)	95.45 (89.71 to 98.51)	N/A
Shah 2020	2680 P	4.14	Moderate DR or worse or CSDME (ICDR & ETDRS)	100 (97 to 100)	81.82 (80 to 83)	No FNs (VTDR was 2.57% of the sample)
van der Heijden 2018	898 P	2.4	Moderate or worse DR or DMO (ICDR & EURODIAB)	ICDR: 68 (56 to 79) EURODIAB: 91 (69 to 98)	ICDR: 86 (84 to 88) EURODIAB: 84 (81 to 86)	1 case of VTDR missed with either of the criteria
Verbraak 2019^j^	1293	5.0	mtmDR or DMO (ICDR)	79.4 (66.5 to 87.9)	93.8 (92.1 to 94.9)	No cases of VTDR were missed
*RedCAD*						
Gonzalez-Gonzalo 2020	1200 Im	41.7	Messidor grades 2 & 3 (Messidor)	92.0 (89.3 to 97.2)	92.1 (88.6 to 95.2)	Not reported
*SELENA (DL)*						
Bellemo 2019	3093 E	22.5 E	Moderate NPDR or worse, or MO or U (ICDR)	92.25 (90.10 to 94.12)	89.04 (87.85 to 90.28)	At RDR threshold SE for VTDR was 99.42% and for DMO 97.19%
Ting 2017^k^	8589 P	3.0 E	Moderate NPDR or worse, or MO or U (ICDR)	89.56 (85.51 to 92.58)	83.49 (82.68 to 84.27)	No cases of VTDR were missed
*Visiona (DL)*						
Ramachandran 2018	294 P	2.7 E	Moderate DR or moderate DMO (NZ MoH)	84.6 (55.0 to 98.0)^b^	79.7 (76.0 to 83.0)^b^	N/A
*Other DL ARIASs*						
Cen 2021^l^	29,343 P	45.7	Moderate NPDR or worse and/or DMO (ICO)	98.1 (98.0 to 98.2)^b^	99.7 (99.6 to 100)^b^	N/A
Kanagasingam 2018	193 P	1.04	Moderate or severe DR (ICDR)	100 (CI not estimable, as only 2 TPs and no FNs)	92 (87 to 96)	No FNs
*iGrading (ML)*						
Fleming 2010a	33,535 P	12.3	M1, M2, R2, R3, R4, U (SDESP)	0.11% (37/33,535) of those requiring referral or 6 month recall were missed	51.7% of those requiring routine 12 month recall were software positive	FNs: M1 (3/387), M2 (31/1130), no R2, R3 or R4 missed
Fleming 2010b	7586	16.5	M1, M2, R2, R3, R4 (SDESP); 2 strategies: (1) MA vs (2) MA + EX + HM	1) 94.9 (93.5 to 96.0) 2) 96.6 (95.4 to 97.4)	51.0 (50.0 to 52.0)^b^	FNs for strategy 2: M1 (8/102), M2 (26/633), R3 (3/272), R4 (6/268)
Goatman 2011	8267 V	7.1	M1, R2, R3, U (EDESP); 4 screening strategies compared: 1. MA, 1 field; 2. MA 2 fields; 3. MA + EX + HM, 1 field; 4. MA + EX + HM, 2 fields	Range: 98.3 (96.9 to 99.1) to 99.3 (98.3 to 99.7), strategy 3 and 2, respectively	Detection rate (range) for: R0: 43.7 to 60.2, R1: 87.6 to 94.8	All strategies detected all R2 and R3; and missed between 1% and 2.5% of M1
Philip 2007^m^	6722	3.8	M1, M2, R1, R2, R3, R4 or U (SDESP)	90.5 (89.3 to 91.6)	67.4 (66.0 to 68.8)	FNs: R1 (232/1640), M1 (2/76), M2 (5/179)
Soto-Pedre 2015^n^	3877	15.6	Any DR (ICDR)	94.52 (92.56 to 96.49)	68.77 (67.18 to 70.36)	All 31 FN cases were mild NPDR
Tufail 2016	20,258	13.7	U, M1, R2, R3 (EDESP)	N/A (failed evaluation)	N/A	N/A
*RetinaLyze (ML)*						
Bouhaimed 2008^o^	96	17.7	Mild NPDR or worse: 2a to 5 (Bro Taf)	88 (64 to 99)	52 (40 to 63)	FNs: 2 patients classified by the RS as 2a (mild NPDR)
Grzybowski 2021^i^	170	45.7	Moderate NPDR or worse and/or DMO (ICO); 2 cutoffs: (a) 1 or (b) 2 positive images for overall positive result	a) 89.66 (78.83 to 96.11) b) 74.14 (60.96 to 84.74)	a) 71.82 (62.44 to 79.98) b) 93.64 (87.33 to 97.40)	N/A
Hansen 2004	83	n/a	Any DR or ungradable; DMO not graded (ETDRS)	No mydriasis: 89.9 (80.0 to 96.0)^b^ Mydriasis: 97.0 (90.0 to 100)^b^	No mydriasis: 85.7 (57.0 to 98.0)^b^ Mydriasis: 75.0 (48.0 to 93.0)^b^	FNs: no mydriasis: 6 mild NPDR and 1 ungradable; mydriasis: 2 mild NPDR
*RetmarkerSR (ML)*						
Figueiredo 2015	11,511 P	6.3	Not reported but reference to the PDESP; 4 datasets	Range: 89.3 to 100	Range: 57.6 to 73.0	N/A
Oliveira 2011^p^	5386 P	8.7	NPDR with DMO or PDR (PDESP)	96.1 (94.39 to 97.89)	51.7 (50.27 to 53.07)	Identified all cases of PDR; missed 0.3% of case with NPDR with maculopathy
Ribeiro 2015	3287 P	2.3	NPDR with MDO or PDR (PDESP)	11 FNs (0.3% of the 3287 quality control cases)	N/A	None of the 11 FN cases were PDR
Tufail 2016	20,258 P	13.7	U, M1, R2, R3 (EDESP)	85.0 (83.6 to 86.2)	47.7 (47 to 48.5)	Identified ∼99% of R2M1, R3M0 and R3M1 and 97% of R2M0

CI: confidence interval; CNDCS: China National Diabetic Complications Study; DESP: Diabetic Eye Screening Programme; DL: deep learning; DMO: diabetic macular oedema; DR: diabetic retinopathy; EDESP: English DESP; ETDRS: Early Treatment of Diabetic Retinopathy Study^61^; EURODIAB: EURODIAB IDDM Complications Study^62^; FN: false negative; ICO: International Council of Ophthalmology; ICDR: International Clinical Diabetic Retinopathy scale^63^; Im: images; ML: machine learning; NPDR: non-proliferative diabetic retinopathy; P: patients; PDR: proliferative diabetic retinopathy; PDESP: Portuguese DESP; RDR: referable diabetic retinopathy; RS: reference standard; SDESP: Scottish DESP; SiDRP: Singapore DESP; SE: sensitivity; SP: specificity; TP: true positive result; U: ungradable; V: visits; WDESP: Welsh DESP.

^a^ Only the dataset from China included as the other 2 datasets did not meet our inclusion criteria.

^b^ Confidence interval not reported in the paper; recalculated from accuracy estimates, proportion of diseased and sample size using Cochrane Collaboration's Review Manager 5.4.1.

^c^ Only the accuracy estimates from the CNDCS 2018 cohort are included here.

^d^ 655 patients is the total of 4 cohorts: 2 sequentially recruited cohorts, 1 in primary care (*n* = 45) and 1 in ophthalmology (*n* = 190); and 2 enrichment permitted cohorts, 1 in primary care (*n* = 335) and 1 in ophthalmology (*n* = 85); hence the range of sensitivities and specificities estimates reported.

^e^ Only the results from standard fundus photography reported here.

^f^ Combined 3 population-based datasets with prevalence of diabetes ranging from 9.4% to 35%; the reported sample size is after excluding 863 eyes due to ungradable or missing reference standard grading.

^g^ Only patient-wise accuracy estimates included here.

^h^ Accuracy estimates reported here reflect the performance of the original model evaluated prospectively; the sensitivity and specificity of the new model evaluated retrospectively on the combined dataset was 92.2% (95% CI, 90.7 to 93.6) and 96.9% (95% CI, 96.2 to 97.5) respectively, for detecting moderate or worse DR.

^i^ Two-gate study including 60 patients with DR and 110 patients with no DR.

^j^ Sample size and accuracy estimates after excluding ‘ungradable’ by RS and device.

^k^ Prevalence based on 14,880 patients of whom 6291 were repeats included in the primary validation dataset; here, we report only the accuracy estimates based on the 8589 patients not included in the development dataset, SiDRP 2014–2015 cohort.

^l^ Only accuracy estimates from the multihospital test cohort are reported here.

^m^ Prevalence is based on the grades considered referable in the SDESP: M2, R2, R3, R4, excluding images classified as ungradable by the RS. The study assessed the accuracy for ‘disease/no-disease’ only, so the definition of RDR is the same as ‘any DR’.

^n^ Estimated prevalence of any DR.

^o^ Only accuracy estimates based on the ‘red & bright lesions’ option of the software are reported; images classified as low quality by the software are included in the analysis.

^p^ Only the accuracy of ‘one-step’ approach (assessment at single time point) included here; the paper also reports on the accuracy of ‘two-step’ approach looking at disease progression between two yearly screens.

RDR covers a range of retinopathy grades, from moderate NPDR which usually requires only close monitoring, to PDR where urgent hospital assessment and treatment might be needed. Since the distribution of grades in the false negatives could vary, sensitivity estimates are insufficient to fully characterise the safety of the system. Hence, we also report the characteristics of the false negatives (Table 3) and the accuracy of the systems for detecting higher grades (Table S8). Most studies reported that at the RDR threshold no higher grades of DR were missed or their proportion was very small; and the sensitivity for higher grades was comparable or exceeded that for RDR. In addition, 13 of the 14 studies that investigated the accuracy of the systems for detecting any DR reported sensitivities ≥85% (Table S8).

Thirty-two studies provided data on ungradable images which were difficult to summarise due to inconsistent reporting. Overall, studies reported high detection rates for low-quality images (as determined by the reference standard) and failed to process only a small proportion of the images deemed gradable. There was some evidence that these aspects of the systems’ performance could be affected by a range of factors, such as differences in the imaging protocols^27,45^ and human behaviour^54^ (Table S8).

Twenty-one studies investigated the impact of various factors on accuracy; they related to patient characteristics (age, sex, ethnicity/race, duration of diabetes and HbA1c); imaging protocol (camera type, number of fields and image resolution), algorithm (lesion types detected by ML systems, DL vs ML) and reference standard (grading criteria and method of adjudication) (Table S8). Only a small number of studies reported on each factor and the results varied across studies. Briefly, the studies reported that sex (6 studies) and mean HbA1c (3 studies) were not associated with accuracy, while age (7 studies), DL vs ML algorithm (1 study), duration of diabetes (1 study), grading criteria (1 study) and the method of adjudication (1 study) were. The rest of the results were inconsistent and varied across studies and systems.

### Test accuracy of ARIASs compared to human graders

Seven studies conducted head-to-head comparison of the accuracy of human graders not involved in the reference grading to that of Google,^31–33^ Selena,^35^ RetCAD,^34^ DLP^36^ and iGradingM^25^ (Table 4). The number of graders was small (range 2-5), except for the study by Raumviboonsuk et al. which included 13 regional graders from the national screening programme in Thailand.^33^ Across all studies ARIASs had higher sensitivity but lower specificity for RDR. In addition, Krause et al. reported that Google had higher sensitivity for referable DMO^32^; and Ting et al. reported that Selena had higher sensitivity but lower specificity for vision-threatening DR.^35^

**Table 4.** Studies comparing directly (in the same sample) the test accuracy of ARIAS and human graders not involved in the reference grading.

ARIAS: study and country	1. Study design 2. Dataset 3. RS	Comparator	Accuracy of human graders, % (95% CI)	Accuracy of ARIAS, % (95% CI)
DLP: Cen 2021, China	Retrospective multi-hospital cohort study 711 images were collected from PACS JSIE Retina expert panel, majority decision	5 retinal specialists, >10 years of clinical experience	Average expert accuracy for RDR: Fundus images only: SE 93.5, SP 99.1 Fundus images + notes: 95.3, SP 99.4	SE 95.1, SP 99.6
Googel AI: Gulshan 2019, India	Prospective cohort study 2 eye care centres in India (>40 years old patients) 3 retinal specialists	1 trained grader and 1 retinal specialist from each site	*RDR* SE ranged from 73.4 to 89.8 SP ranged from 83.5 to 98.7 *DMO* SE ranged from 57.5 to 89.5 SP ranged from 93.8 to 99.3	*RDR (site 1 & 2, respectively)* SE 88.9, 92.1 SP 92.2, 95.2 *DMO (site 1 & 2, respectively)* SE 97.4, 93.6 SP 90.7, 92.5
Google AI: Krause 2018, USA	Retrospective cohort study EyePACS-2: 1958 images from 998 unique individuals Consensus by 3 retinal specialists	3 ophthalmologists, individually and as a majority decision	*Accuracy for RDR*: Ophthalmologists’ majority decision: SE 83.8 SP 98.1 Individual ophthalmologists (range): SE 74.9 to 76.4, SP 97.5 to 99.1 *Accuracy for referable DMO*: Ophthalmologists’ majority decision: SE 83.3, SP 99.0 Individual ophthalmologists (range): SE 62.7 to 86.4, SP 98.6 to 99.1	*Accuracy for RDR*: SE 97.1 SP 92.3 *Accuracy for referable DMO*: SE 94.9 SP 94.4 No statistical comparison reported
Google AI: Raumviboonsuk 2019, Thailand	Retrospective cohort study Diabetes registry Partially adjudicated by independent retinal specialists	13 regional graders from Thailand DESP (7 ophthalmologists and 6 trained ophthalmic nurses or technicians; >2 years of experience)	*Accuracy for RDR* SE 73.4 (range: 40.7 to 91.4 across regional graders) SP 98.0 (range: 93.9 to 100) *Accuracy for DMO* SE 62.0 (range: 45.0 to 80.3) SP 99.2 (range: 97.3 to 99.8) *Accuracy for severe NPDR/worse* AUC 0.993 (range: 0.974 to 0.995)	*Accuracy for RDR* SE 96.8 (range: 89.3 to 99.3) (difference 24%, *p* < 0.001) SP 95.6 (range: 98.3 to 98.7) (difference -2.5%, *p* < 0.001) *Accuracy for DMO* SE 95.3 (range: 85.9 to 100) SP 92.2 (range: 94.4 to 99.1) *Accuracy for severe NPDR/worse* AUC 0.991 (range: 0.978 to 0.997)
iGradingM/Aberdeen system: Philip 2007, UK	Prospective cohort study 14,406 images from 6722 consecutive patients from the SDESP A single clinical research fellow	3 retinal screeners who also performed the photography	*Technical failures*: SE 93.7 (91.3 to 95.4) SP 99.0 (98.7 to 99.2) *Accuracy for RDR*: SE 86.5 (85.1 to 87.8) SP 95.3 (94.6 to 95.9) *N of patients* misclassified as ‘no DR’: 341 *N of patients* with M1, R2, M2, R3 or R4 graded as ‘no DR’: 3/330	*Technical failures*: SE 99.5 (98.4 to 99.8) SP 84.4 (83.5 to 85.3) *Accuracy for RDR*: SE 90.5 (89.3 to 91.6) SP 67.4 (66.0 to 68.8) *N of patients* misclassified as ‘no DR’: 240, *p* < 0.001 *N of patients* with M1, R2, M2, R3 or R4 graded as ‘no DR’: 7/330, *p* = 0.125
RetCAD: Gonzalez-Gonzalo 2020, Spain, The Netherlands	Retrospective cohort study Messidor (n = 1200) Dataset's ground truth	2 graders, a general ophthalmologist and a retinal specialist, with 4 and 20 years of DR screening experience, respectively	*Accuracy for RDR*: HG1 SE 79.6 (74.8 to 84.8) SP 97.7 (96.0 to 99.2) HG2 SE 69.0 (62.9 to 74.7) SP 99.1 (97.9 to 100)	*Accuracy for RDR* SE 92.0 (89.1 to 95.9) SP 92.1 (88.7 to 95.2) No statistical comparison reported
SELENA: Ting 2017, Singapore	Retrospective cohort study 8589 unique patients (excluding those used in the development) A single retinal specialist with >5 years experience	2 trained senior nonmedical professional graders with >5 years experience, currently employed in the SiDRP	*Accuracy for RDR*: SE 84.84 (81.28 to 88.51) SP 98.55 (98.27 to 98.79) *Accuracy for VTDR*: SE 89.74 (74.77 to 96.27) SP 99.09 (98.86 to 99.27)	*Accuracy for RDR*: SE 89.56 (85.51 to 92.58), *p* = 0.04 SP 83.49 (82.68 to 84.27), *p* < 0.001 *Accuracy for VTDR*: SE 100 (90.97 to 100), *p* = 0.04 SP 81.4 (80.57 to 82.22), *p* < 0.001

ARIAS: artificial intelligence-based automated retinal imaging assessment system; CI: confidence interval; DESP: Diabetic Eye Screening Programme; DMO: diabetic macular oedema; DR: diabetic retinopathy; HG1: Human Grader 1; HG2: Human Grader 2; RDR: referable diabetic retinopathy; RS: reference standard; SE: sensitivity; SDESP: Scottish DESP; SiDRP: Singapore DESP; SP: specificity; VTDR: vision-threatening diabetic retinopathy.

## Discussion

The evaluation of the accuracy of AI-based medical tests goes through a number of stages. Prior to deployment, the systems should be evaluated in the target clinical pathway or a similar setting, so that the accuracy estimates could be used in clinical and policy decisions.^59^ In the current paper, we reviewed the accuracy of ARIASs that have been approved for clinical use, are commercially available or have had at least one evaluation in a clinically plausible cohort of patients. Accuracy is the primary, albeit not the only, aspect of such systems’ performance. Unbiased and applicable evidence of acceptable accuracy should be the starting point when considering ARIASs for implementation in clinical practice.

We included 43 studies evaluating 15 DL-based and 4 traditional ML-based systems. Most studies reported sensitivity ≥85% for RDR, any DR and higher grades. At the RDR threshold no cases of proliferative/treatable disease were missed or their proportion was very small. Specificity estimates, on the other hand, varied considerably but, overall, DL systems had higher specificity than ML systems. Seven studies reported that ARIASs had higher sensitivities but lower specificities compared to human graders not involved in the reference grading. In addition, most studies reported high accuracy for detection of ungradable images as determined by the reference standard (also referred to as ‘imageability’), and a small proportion of cases where the system was unable to read an image classified as gradable by the reference standard (‘technical failure’).

The above results suggest that with respect to missing cases of severe or treatable disease, ARIASs could be safe to use in clinical practice provided the operational environment is similar to that in which the system has been evaluated. Limited data suggest that various factors could affect the accuracy of ARIASs for detection of DR and images of low quality as well as the proportion of technical failures. The variability in results indicates that such associations are likely to be system- and context-specific and should be investigated prior to deployment in the target clinical pathway.

Ultimately, the level of accuracy required will depend on the specific role of the system in the clinical pathway. For instance, when used in organised screening to rule out DR prior to manual grading (e.g. UK DESPs), lower specificity might not be an issue. Tufail et al. showed that in the English DESP, ARIASs with high sensitivity and specificity as low as 20% are still cost-effective both as a replacement of level 1 graders and when added to the existing clinical pathway prior to manual grading.^27^ In addition, such programmes have well-established quality assurance systems that would allow monitoring and prompt action if there is a decline in the performance of ARIAS over time. Audit studies from Scotland and Portugal provide some real-life evidence that in such settings ARIASs with high sensitivity and relatively low specificity lead to a reduction in the human graders’ workload without compromising the safety of the screening programme.^12,13^

In settings where such quality controls are not readily available, low specificity (including technical failures) is likely to increase the number of unnecessary referrals and the associated costs. Of particular importance here are contextual and human factors as illustrated by van der Heijden et al. who assessed the accuracy of an ARIAS in a primary care setting in the Netherlands. The authors report a high number of referrals due to ‘insufficient quality’ because clinicians underutilised the ‘insufficient quality’ function of the system and preferred to make a referral instead of repeating the imaging using dilation.^54^

Despite these promising results, the evidence base has several limitations. Firstly, most ARIASs were evaluated only in 1–2 studies and none of those evaluations were carried out independently from the developer/manufacturer of the system. The few systems evaluated in multiple studies provide evidence that the accuracy of ARIASs could be affected by multiple factors, especially in terms of specificity. This means that an evaluation in the target clinical pathway is necessary even when there is robust evidence of high performance from another context. Secondly, although the consistent high sensitivity across studies is reassuring, between-study comparison of alternative systems is not possible due to considerable differences in study design. For instance, 85% sensitivity when the reference standard involves a combination of mydriatic 4-wide field stereo retinal photography and optical coherence tomography is not the same as 85% sensitivity when the reference standard is based on the same standard 45° fundus photographs used with the index test. Thirdly, many of the studies were judged to be at high risk of bias in at least one QUADAS-2 domain. Of particular concern is the risk of selection bias, both at patient and image level, and the exclusion of ungradable images. This could lead to inflated accuracy estimates and decline in the system's performance when deployed in clinical practice. Detailed reporting of the selection process, both at patient and image level, and reporting of ungradable images in a way that makes it easier to evaluate their impact are highly desirable and will significantly improve the usability of the evidence.

Direct evaluation of the clinical effectiveness and overall impact of an ARIAS using randomised controlled trial (RCT) designs might not always be feasible. Hence, the importance of assessing the accuracy of the system in a representative dataset, both at patient and image level, and in real-life conditions. Such pre-implementation assessment should also provide evidence on other aspects of the system's performance, such as interoperability, reliability and acceptability. Then a linked evidence approach could be used to combine the evidence and estimate the clinical and cost-effectiveness of an ARIAS even in the absence of RCTs.^60^

The following methodological limitations of the review should be acknowledged: we included only peer-reviewed, English-language journal articles; only 20% of the titles/abstracts and full texts were double-screened; only 20% of the extracted data and methodological quality decisions were verified by a second reviewer; some of the inclusion/exclusion criteria were modified after publication of the protocol.

## Conclusion

Across systems and studies, ARIASs had ≥85% sensitivity for RDR and higher grades, higher than human graders, and could be safe to use in clinical practice (i.e. have a low risk of missing proliferative/treatable DR) provided the operational environment in the target clinical pathway is similar to that in which the system has been evaluated. The specificities were much more variable, even across studies evaluating the same system. Relatively low specificity might be acceptable when the system is used prior to manual grading in organised screening with established quality assurance protocols. In other settings, however, low specificity is likely to generate unnecessary referrals and incur additional costs. Since the accuracy and overall performance of ARIASs might be affected by a wide range of factors, including human behaviour, evaluation in the target clinical pathway prior to deployment is advisable. If an RCT is not feasible, the clinical and cost-effectiveness of the system relative to the current clinical pathway could be assessed using a linked evidence approach. This however will require not only unbiased and applicable accuracy estimates, but also evaluation of other aspects of the system's performance, such as interoperability, reliability and acceptability.

## Supplemental Material

sj-docx-1-msc-10.1177_09691413221144382 - Supplemental material for Test accuracy of artificial intelligence-based grading of fundus images in diabetic retinopathy screening: A systematic review Click here for additional data file. Supplemental material, sj-docx-1-msc-10.1177_09691413221144382 for Test accuracy of artificial intelligence-based grading of fundus images in diabetic retinopathy screening: A systematic review by Zhivko Zhelev, Jaime Peters, Morwenna Rogers, Michael Allen, Goda Kijauskaite, Farah Seedat, Elizabeth Wilkinson and Christopher Hyde in Journal of Medical Screening
