# Supplementary material for: Test accuracy of artificial intelligence-based grading of fundus images in diabetic retinopathy screening: A systematic review
Source: J Med Screen. 2023 Jan 9;30(3):97–112. doi: 10.1177/09691413221144382 (PMC10399100; doi:10.1177/09691413221144382)
Supplement: sj-docx-1-msc-10.1177_09691413221144382 - Supplemental material for Test accuracy of artificial intelligence-based grading of fundus images in diabetic retinopathy screening: A systematic review [file sj-docx-1-msc-10.1177_09691413221144382.docx]

**Test accuracy of artificial intelligence-based grading of fundus images in diabetic retinopathy screening: A systematic review**

**Zhelev Z, Peters J, Rogers M, Allen M, Kijauskaite G, Seedat F, Wilkinson E, Hyde C**

**Supplementary material**

**Table of contents:**

**Figure S1 Flow diagram of the selection of studies……………………………………………………………1**

**Table S1 Search strategy………………………………………………………………………………………..2**

**Table S2 QUADAS-2 criteria…………………………………………………………………………………...3**

**Table S3 QUADAS-2C criteria…………………………………………………………………………………3**

**Table S4 Studies excluded at full text with reason for exclusion……………………………………………..4**

**Table S5 Included ARIASs and number of studies evaluating each system………………………………..17**

**Table S6 For each AI-system, the table shows study-level sensitivity, specificity, PPV and NPV arranged by prevalence in ascending order………………………………………………………………………………17**

**Table S7 The table shows study-level sensitivity, specificity, PPV and NPV arranged by prevalence and grouped by 10% increments……………………………………………………………………………………18**

**Table S8 Test accuracy at other thresholds and factors affecting accuracy………………………………..20**

**Figure S1 Flow diagram of the selection of studies**

**
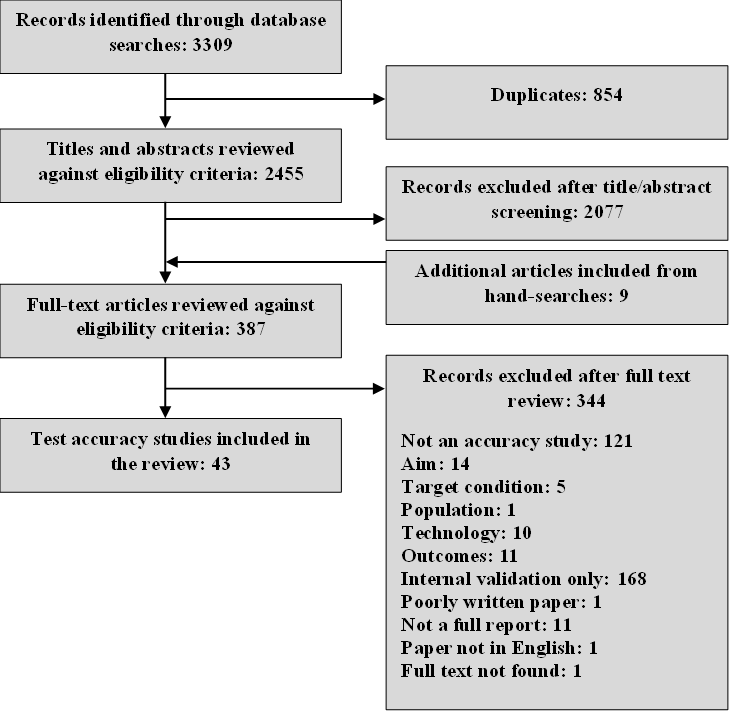
**

**Table S1 Search strategy for Ovid MEDLINE(R) ALL <1946 to June 25, 2020>**

| N Terms (N of hits) |
| --- |
| 1 exp eye diseases/ (563274) |
| 2 retinopathy.ti,ab. (43575) |
| 3 eye pathology.ti,ab. (319) |
| 4 maculopathy.ti,ab. (4228) |
| 5 diabetic eye.ti,ab. (732) |
| 6 diabetic macular.ti,ab. (4004) |
| 7 retinal fundus.ti,ab. (315) |
| 8 1 or 2 or 3 or 4 or 5 or 6 or 7 (578122) |
| 9 exp Diagnostic Techniques, Ophthalmological/ (169047) |
| 10 exp Diagnosis, Computer-Assisted/ (83108) |
| 11 "Sensitivity and Specificity"/ (346232) |
| 12 diagnostic test*.ti,ab. (46123) |
| 13 diagnostic accuracy.ti,ab. (43812) |
| 14 diagnostic performance.ti,ab. (16194) |
| 15 screening.ti,ab. (520833) |
| 16 imaging.ti,ab. (798701) |
| 17 (Sensitivity or specificity).ti,ab. (1033830) |
| 18 reference standard.ab. (14139) |
| 19 optical coherence tomography.ti,ab. (35177) |
| 20 or/9-19 (2600413) |
| 21 exp Artificial Intelligence/ (96870) |
| 22 artificial intelligence.ti,ab. (6988) |
| 23 deep learning.ti,ab. (8539) |
| 24 neural network*.ti,ab. (48131) |
| 25 automated retinal image analysis system.ti,ab. (2) |
| 26 automated grading.ti,ab. (72) |
| 27 automated level.ti,ab. (8) |
| 28 (automated adj (tool* or technique* or identification or detection)).ti,ab. (4376) |
| 29 ARIAS.ti,ab. (299) |
| 30 iGradingM.ti,ab. (2) |
| 31 EyeArt.ti,ab. (5) |
| 32 IDx-DR.ti,ab. (6) |
| 33 DR-RACS.ti,ab. (0) |
| 34 RetinaLyze.ti,ab. (1) |
| 35 RetmarkerSR DR.ti,ab. (0) |
| 36 Singapore Eye Lesion Analyzer.ti,ab. (0) |
| 37 RetinaVue.ti,ab. (0) |
| 38 TRIAD network.ti,ab. (0) |
| 39 or/21-38 (135626) |
| 40 8 and 20 and 39 (1048) |
| 41 limit 40 to yr="2000 -Current" (991) |
|  |

**Table S2 QUADAS-2 criteria**

| **Domain** | **Signalling questions** |
| --- | --- |
| Patient selection | 1. Was a consecutive or random sample of patients enrolled? Yes, if clear from the paper  2. Was a case-control design avoided? Yes, if clear from the paper  3. Did the study avoid inappropriate exclusions? Yes, if no patients that would normally be included in diabetic retinopathy screening were excluded. (*If no patients that would normally be included in the English DESP were excluded)  Risk of bias: Low if all of the above are answered ‘yes’  Applicability concerns: Low if patients with type 1 or type 2 diabetes eligible for diabetic retinopathy screening were included (*Low only if the study is conducted in the relevant UK population; Unclear, if conducted in a non-UK population, unless there is a clear indication that the population is different from the target population, e.g. a mixture of diabetic and non-diabetic patients) |
| Index test | 1. Were the index test results interpreted without knowledge of the results of the reference standard? In most cases, this will be ‘yes’, unless specific reason is given  2. If a threshold was used, was it pre-specified? Yes, if clear from the paper  Risk of bias: Low, if all of the above are answered ‘yes’  Applicability concerns: Low if in line with usual practice of diabetic retinopathy screening (*High, if different from the intended EDESP use of the system) |
| Reference standard | 1. Is the reference standard likely to classify correctly the target condition? Yes, the reference standard involves a panel of retinal specialists, ophthalmologists or similar experts independently reading the images and resolving disagreements according to a pre-specified protocol; Yes, if the final grades from a national screening programme with multilevel grading have been used as a ground truth; No, if a single grader determines the ground truth.  2. Were the reference standard results interpreted without knowledge of the results of the index test? Yes, if clear from the paper  Risk of bias: Low, if all of the above are answered ‘yes’  Applicability concerns: High, if the definition of DR is different from the one used in the EDESP, e.g. maculopathy is not included (*No changes made to the original criterion). |
| Flow and timing | 1. Did all patients receive a reference standard?  2. Did all patients receive the same reference standard?  3. Were all patients included in the analysis? No, if ungradable images have been excluded.  Risk of bias: Low, if all of the above are answered ‘yes’ |
| *Definition as used in the original analysis or a note that the original definition has not been changed  EDESP – English Diabetic Eye Screening Programme | |

**Table S3 QUADAS-2C criteria**

| Domain | Signalling questions |
| --- | --- |
| Patient selection* | C1.1 Was the risk of bias for this domain judged ‘low’ for all index tests?  C1.2 Was the intention for patients either to receive all index tests or to be randomly allocated to index tests?  C1.3 If patients were randomized, was the allocation sequence random?  C1.4 If patients were randomized, was the allocation sequence concealed until patients were enrolled and assigned to index tests? |
| Index test | C2.1 Was the risk of bias for this domain judged ‘low’ for all index tests?  C2.2 If patients received multiple index tests, were test results interpreted without knowledge of the results of the other index test(s)?  C2.3 If patients received multiple index tests, is undergoing one index test unlikely to affect the performance of the other index test(s)?  C2.4 Were differences in the conduct or interpretation between the index tests unlikely to advantage one of the tests? |
| Reference standard | C3.1 Was the risk of bias for this domain judged ‘low’ for all index tests?  C3.2 Did the reference standard avoid incorporating any of the index tests? |
| Flow and timing | C4.1 Was the risk of bias for this domain judged ‘low’ for all index tests?  C4.2 Was there an appropriate interval between the index tests?  C4.3 Was the same reference standard used for all index tests?  C4.4 Are the proportions and reasons for missing data similar across index tests?  C4.5 Could the patient flow have introduced bias in the comparison? |

**Table S4 List of studies excluded at full text with reason for exclusion**

| **Study** | **Reason for exclusion** |
| --- | --- |
| Abbas Q, Fondon I, Sarmiento A, Jimenez S, Alemany P. Automatic recognition of severity level for diagnosis of diabetic retinopathy using deep visual features. Med Biol Eng Comput. 2017;55(11):1959-74. | Internal validation only |
| Abidalkareem AJ, Abd MA, Ibrahim AK, Zhuang H, Altaher AS, Muhamed Ali A. Diabetic Retinopathy (DR) Severity Level Classification Using Multimodel Convolutional Neural Networks. Annual International Conference of the IEEE Engineering in Medicine and Biology Society IEEE Engineering in Medicine and Biology Society Annual International Conference. 2020;2020:1404-7. | Internal validation only |
| Abramoff MD, Folk JC, Han DP, Walker JD, Williams DF, Russell SR, et al. Automated analysis of retinal images for detection of referable diabetic retinopathy. JAMA Ophthalmology. 2013;131(3):351-7. | Technology |
| Abramoff MD, Leng T, Ting DSW, Rhee K, Horton MB, Brady CJ, et al. Automated and Computer-Assisted Detection, Classification, and Diagnosis of Diabetic Retinopathy. Telemedicine journal and e-health : the official journal of the American Telemedicine Association. 2020;26(4):544-50. | Not a DTA study |
| Abramoff MD, Niemeijer M, Russell SR. Automated detection of diabetic retinopathy: barriers to translation into clinical practice. Expert Rev Med Devices. 2010;7(2):287-96. | Not a DTA study |
| Abramoff MD, Niemeijer M, Suttorp-Schulten MS, Viergever MA, Russell SR, van Ginneken B. Evaluation of a system for automatic detection of diabetic retinopathy from color fundus photographs in a large population of patients with diabetes. Diabetes Care. 2008;31(2):193-8. | Not a DTA study |
| Abramoff MD, Tobey D, Char DS. Lessons Learned About Autonomous AI: Finding a Safe, Efficacious, and Ethical Path Through the Development Process. Am J Ophthalmol. 2020;214:134-42. | Not a DTA study |
| Acharya UR, Lim CM, Ng EY, Chee C, Tamura T. Computer-based detection of diabetes retinopathy stages using digital fundus images. Proc Inst Mech Eng [H]. 2009;223(5):545-53. | Internal validation only |
| Acharya UR, Mookiah MRK, Koh JEW, Tan JH, Bhandary SV, Rao AK, et al. Automated diabetic macular oedema (DME) grading system using DWT, DCT Features and maculopathy index. Comput Biol Med. 2017;84:59-68. | Internal validation only |
| Acharya UR, Ng EY, Tan JH, Sree SV, Ng KH. An integrated index for the identification of diabetic retinopathy stages using texture parameters. Journal of Medical Systems. 2012;36(3):2011-20. | Internal validation only |
| Adal KM, Sidibe D, Ali S, Chaum E, Karnowski TP, Meriaudeau F. Automated detection of microaneurysms using scale-adapted blob analysis and semi-supervised learning. Computer Methods and Programs in Biomedicine. 2014;114(1):1-10. | Internal validation only |
| Adal KM, van Etten PG, Martinez JP, Rouwen KW, Vermeer KA, van Vliet LJ. An Automated System for the Detection and Classification of Retinal Changes Due to Red Lesions in Longitudinal Fundus Images. IEEE Trans Biomed Eng. 2018;65(6):1382-90. | Aim |
| Akbar S, Akram MU, Sharif M, Tariq A, Yasin UU. Decision Support System for Detection of Papilledema through Fundus Retinal Images. Journal of Medical Systems. 2017;41(4):66. | Target condition |
| Akram MU, Tariq A, Anjum MA, Javed MY. Automated detection of exudates in colored retinal images for diagnosis of diabetic retinopathy. Appl Opt. 2012;51(20):4858-66. | Internal validation only |
| Akram MU, Tariq A, Khan SA, Javed MY. Automated detection of exudates and macula for grading of diabetic macular edema. Comput Methods Programs Biomed. 2014;114(2):141-52. | Internal validation only |
| Akram UM, Khan SA. Automated detection of dark and bright lesions in retinal images for early detection of diabetic retinopathy. Journal of Medical Systems. 2012;36(5):3151-62. | Internal validation only |
| Akyol K, Sen B, Bayir S. Automatic Detection of Optic Disc in Retinal Image by Using Keypoint Detection, Texture Analysis, and Visual Dictionary Techniques. Comput. 2016;2016:6814791. | Internal validation only |
| Alam M, Le D, Lim JI, Chan RVP, Yao X. Supervised Machine Learning Based Multi-Task Artificial Intelligence Classification of Retinopathies. Journal of Clinical Medicine. 2019;8(6):18. | Technology |
| Alexander A, Jiang A, Ferreira C, Zurkiya D. An Intelligent Future for Medical Imaging: A Market Outlook on Artificial Intelligence for Medical Imaging. J. 2020;17(1 Pt B):165-70. | Not a DTA study |
| Al-Jarrah MA, Shatnawi H. Non-proliferative diabetic retinopathy symptoms detection and classification using neural network. J Med Eng Technol. 2017;41(6):498-505. | Internal validation only |
| Alqudah AM. AOCT-NET: a convolutional network automated classification of multiclass retinal diseases using spectral-domain optical coherence tomography images. Med Biol Eng Comput. 2020;58(1):41-53. | Technology |
| Al-Rawi M, Karajeh H. Genetic algorithm matched filter optimization for automated detection of blood vessels from digital retinal images. Computer Methods and Programs in Biomedicine. 2007;87(3):248-53. | Internal validation only |
| Amin J, Sharif M, Rehman A, Raza M, Mufti MR. Diabetic retinopathy detection and classification using hybrid feature set. Microsc Res Tech. 2018;81(9):990-6. | Internal validation only |
| Andreasen M, Kjellberg J. A health economic analysis: RetinaLyzeTM . The Danish Committee for Health Education. December 2008 | Not a DTA study |
| Anitha J, Vijila CK, Selvakumar AI, Indumathy A, Jude Hemanth D. Automated multi-level pathology identification techniques for abnormal retinal images using artificial neural networks. British Journal of Ophthalmology. 2012;96(2):220-3. | Internal validation only |
| Anonymous. All eyes are on AI. Nat. 2018;2(3):139. | Not a DTA study |
| Anonymous. Ascent of machine learning in medicine. Nat Mater. 2019;18(5):407. | Not a DTA study |
| Araujo T, Aresta G, Mendonca L, Penas S, Maia C, Carneiro A, et al. DR\|GRADUATE: Uncertainty-aware deep learning-based diabetic retinopathy grading in eye fundus images. Med Image Anal. 2020;63:101715 | Outcomes |
| Arcadu F, Benmansour F, Maunz A, Michon J, Haskova Z, McClintock D, et al. Deep Learning Predicts OCT Measures of Diabetic Macular Thickening From Color Fundus Photographs. Invest Ophthalmol Vis Sci. 2019;60(4):852-7. | Internal validation only |
| Armstrong S. The computer will assess you now. Bmj. 2016;355:i5680. | Not a DTA study |
| Arsalan M, Owais M, Mahmood T, Cho SW, Park KR. Aiding the Diagnosis of Diabetic and Hypertensive Retinopathy Using Artificial Intelligence-Based Semantic Segmentation. Journal of Clinical Medicine. 2019;8(9):11. | Internal validation only |
| Arunkumar R, Balakrishnan N. Medical image classification for disease diagnosis by DBN methods. Pakistan Journal of Biotechnology. 2018;15(1):107-10. | Not a DTA study |
| Ayhan MS, Kuhlewein L, Aliyeva G, Inhoffen W, Ziemssen F, Berens P. Expert-validated estimation of diagnostic uncertainty for deep neural networks in diabetic retinopathy detection. Med Image Anal. 2020;64:101724. | Not a DTA study |
| Badar M, Haris M, Fatima A. Application of deep learning for retinal image analysis: A review. Computer Science Review Volume 35, February 2020 | Not a DTA study |
| BahadarKhan K, Khaliq AA, Shahid M. A morphological hessian based approach for retinal blood vessels segmentation and denoising using region based otsu thresholding. PLoS ONE. 2016;11 (7) (no pagination)(e0158996). | Internal validation only |
| Bala MP, Vijayachitra S. Early detection and classification of microaneurysms in retinal fundus images using sequential learning methods. International Journal of Biomedical Engineering and Technology. 2014;15(2):128-43. | Internal validation only |
| Balachandar N, Chang K, Kalpathy-Cramer J, Rubin DL. Accounting for data variability in multi-institutional distributed deep learning for medical imaging. Journal of the American Medical Informatics Association : JAMIA. 2020;27(5):700-8. | Aim |
| Balyen L, Peto T. Promising Artificial Intelligence-Machine Learning-Deep Learning Algorithms in Ophthalmology. Asia Pac J Ophthalmol (Phila). 2019;8(3):264-72. | Not a DTA study |
| Banerjee S, Kayal D. Detection of hard exudates using mean shift and normalized cut method. Biocybernetics and Biomedical Engineering. 2016;36(4):679-85. | Internal validation only |
| Banuselvasaraswathy B, Arul Murugan C, Karthigaikumar P. Automatic retinal lesions detection of diabetic retinopathy using curvelet based enhancement. Indian Journal of Public Health Research and Development. 2019;10(2):1029-35. | Internal validation only |
| Basu S, Johnson KT, Berkowitz SA. Use of Machine Learning Approaches in Clinical Epidemiological Research of Diabetes. Current diabetes reports. 2020;20(12):80. | Not a DTA study |
| Bellemo V, Lim G, Rim TH, Tan GSW, Cheung CY, Sadda S, et al. Artificial Intelligence Screening for Diabetic Retinopathy: the Real-World Emerging Application. Curr Diab Rep. 2019;19(9):72. | Not a DTA study |
| Berens P, Waldstein SM, Ayhan MS, Kummerle L, Agostini H, Stahl A, et al. [Potential of methods of artificial intelligence for quality assurance]. Ophthalmologe. 2020;117(4):320-5. | Paper not in English |
| Bhaskaranand M, Ramachandra C, Bhat S, Solanki K. Cost savings enabled by automated diabetic retinopathy screening in a UK-like screening program. Investigative Ophthalmology and Visual Science. 2016;57 (12):5584. | Not a DTA study |
| Bhuiyan A, Govindaiah A, Deobhakta A, Gupta M, Rosen R, Saleem S, et al. Development and Validation of an Automated Diabetic Retinopathy Screening Tool for Primary Care Setting. Diabetes care. 2020;43(10): e147-e148. | Not a full report |
| Bora A, Babenko B, Varadarajan AV, Virmani S, Cuadros J, Balasubramanian S. Deep Learning for Predicting the Progression of Diabetic Retinopathy using Fundus Images. Investigative Ophthalmology and Visual Science. 2020;61(7). | Not a full report |
| Boucher MC, Qian J, Brent MH, Wong DT, Sheidow T, Duval R, et al. Evidence-based Canadian guidelines for tele-retina screening for diabetic retinopathy: recommendations from the Canadian Retina Research Network (CR2N) Tele-Retina Steering Committee. Can J Ophthalmol. 2020;55(1S1):14-24. | Not a DTA study |
| Bourla A, Ferreri F, Ogorzelec L, Peretti CS, Guinchard C, Mouchabac S. Psychiatrists' Attitudes Toward Disruptive New Technologies: Mixed-Methods Study. JMIR Ment Health. 2018;5(4):e10240. | Not a DTA study |
| Broome DT, Hilton CB, Mehta N. Policy Implications of Artificial Intelligence and Machine Learning in Diabetes Management. Curr Diab Rep. 2020;20(2):5. | Not a DTA study |
| Buchanan CR, Trucco E. Contextual detection of diabetic pathology in wide-field retinal angiograms. Conf Proc IEEE Eng Med Biol Soc. 2008;2008:5437-40. | Internal validation only |
| Caixinha M, Nunes S. Machine Learning Techniques in Clinical Vision Sciences. Curr Eye Res. 2017;42(1):1-15. | Not a DTA study |
| Cao K, Xu J, Zhao WQ. Artificial intelligence on diabetic retinopathy diagnosis: an automatic classification method based on grey level co-occurrence matrix and naive Bayesian model. International Journal of Ophthalmology. 2019;12(7):1158-62. | Internal validation only |
| Cao P, Ren F, Wan C, Yang J, Zaiane O. Efficient multi-kernel multi-instance learning using weakly supervised and imbalanced data for diabetic retinopathy diagnosis. Comput Med Imaging Graph. 2018;69:112-24. | Internal validation only |
| Cao W, Czarnek N, Shan J, Li L. Microaneurysm Detection Using Principal Component Analysis and Machine Learning Methods. IEEE Trans Nanobioscience. 2018;17(3):191-8. | Outcomes |
| Carter S, Win K, Wang L, Rogers W, Richards B, Houssami N. Ethical, legal and social implications of artificial intelligence systems for screening and diagnosis. BMJ Evidence-Based Medicine. 2019;24 (Supplement 2):A37-A8. | Not a DTA study |
| Channa R, Wolf R, Abramoff MD. Autonomous Artificial Intelligence in Diabetic Retinopathy: From Algorithm to Clinical Application. J Diabetes Sci Technol. 2020:1932296820909900. | Not a DTA study |
| Chee RI, Darwish D, Fernandez-Vega A, Patel S, Jonas K, Ostmo S, et al. Retinal Telemedicine. Current Ophthalmology Reports. 2018;6(1):36-45. | Not a DTA study |
| Chen PC, Liu Y, Peng L. How to develop machine learning models for healthcare. Nat Mater. 2019;18(5):410-4. | Not a DTA study |
| Cheung CY, Tang F, Ting DSW, Tan GSW, Wong TY. Artificial Intelligence in Diabetic Eye Disease Screening. Asia Pac J Ophthalmol (Phila). 2019;24:24. | Not a DTA study |
| Chi CS. Deep learning based automated detection and grading of diabetic retinopathy for screening programme. http://wwwwhoint/trialsearch/Trial2aspx?TrialID=ChiCTR-SON-17010692. 2017. | Not a DTA study |
| Choi JY, Yoo TK, Seo JG, Kwak J, Um TT, Rim TH. Multi-categorical deep learning neural network to classify retinal images: A pilot study employing small database. PLoS ONE. 2017;12 (11) (no pagination)(e0187336). | Internal validation only |
| Chowriappa P, Dua S, Rajendra Acharya U, Muthu Rama Krishnan M. Ensemble selection for feature-based classification of diabetic maculopathy images. Comput Biol Med. 2013;43(12):2156-62. | Internal validation only |
| Chudzik P, Al-Diri B, Caliva F, Ometto G, Hunter A. Exudates Segmentation using Fully Convolutional Neural Network and Auxiliary Codebook. Conf Proc IEEE Eng Med Biol Soc. 2018;2018:770-3. | Internal validation only |
| Chudzik P, Majumdar S, Caliva F, Al-Diri B, Hunter A. Microaneurysm detection using fully convolutional neural networks. Comput Methods Programs Biomed. 2018;158:185-92. | Internal validation only |
| Colomer A, Igual J, Naranjo V. Detection of Early Signs of Diabetic Retinopathy Based on Textural and Morphological Information in Fundus Images. Sensors (Basel, Switzerland). 2020;20(4). | Internal validation only |
| Coppola F, Faggioni L, Regge D, Giovagnoni A, Golfieri R, Bibbolino C, et al. Artificial intelligence: radiologists' expectations and opinions gleaned from a nationwide online survey. Radiol Med (Torino). 2020;29:29. | Not a DTA study |
| Coyner AS, Campbell JP, Chiang MF. Demystifying the Jargon: The Bridge between Ophthalmology and Artificial Intelligence. Ophthalmol Retina. 2019;3(4):291-3. | Not a DTA study |
| Cuadros J. The Real-World Impact of Artificial Intelligence on Diabetic Retinopathy Screening in Primary Care. J Diabetes Sci Technol. 2020:1932296820914287. | Not a DTA study |
| Dai L, Fang R, Li H, Hou X, Sheng B, Wu Q, et al. Clinical Report Guided Retinal Microaneurysm Detection With Multi-Sieving Deep Learning. IEEE Trans Med Imaging. 2018;37(5):1149-61. | Internal validation only |
| De Fauw J, Keane P, Tomasev N, Visentin D, van den Driessche G, Johnson M, et al. Automated analysis of retinal imaging using machine learning techniques for computer vision. F1000Res. 2016;5:1573. | Not a DTA study |
| De Fauw J, Ledsam JR, Romera-Paredes B, Nikolov S, Tomasev N, Blackwell S, et al. Clinically applicable deep learning for diagnosis and referral in retinal disease. Nat Med. 2018;24(9):1342-50. | Technology |
| Dharmawan DA, Boon Poh N. A new two-dimensional matched filter based on the modified Chebyshev type I function for retinal vessels detection. Conf Proc IEEE Eng Med Biol Soc. 2017;2017:369-72. | Internal validation only |
| Di X, Shuang Y, Vignarajan J, Dong A, Mei-Ling T-K, Kanagasingam Y. Retinal hemorrhage detection by rule-based and machine learning approach. Conf Proc IEEE Eng Med Biol Soc. 2017;2017:660-3. | Internal validation only |
| Dismuke C. Progress in examining cost-effectiveness of AI in diabetic retinopathy screening. The Lancet Digital Health. 2020;2(5):e212-e3. | Not a DTA study |
| Dou X-Y, Xiao L-L. Application of artificial intelligence and deep learning in opthalmology. International Eye Science. 2020;20(7):1197-201. | Not a DTA study |
| Dupas B, Walter T, Erginay A, Ordonez R, Deb-Joardar N, Gain P, et al. Evaluation of automated fundus photograph analysis algorithms for detecting microaneurysms, haemorrhages and exudates, and of a computer-assisted diagnostic system for grading diabetic retinopathy. Diabetes Metab. 2010;36(3):213-20. | Internal validation only |
| Eftekhari N, Pourreza HR, Masoudi M, Ghiasi-Shirazi K, Saeedi E. Microaneurysm detection in fundus images using a two-step convolutional neural network. Biomedical Engineering Online. 2019;18(1):67. | Internal validation only |
| Egan C, Rudisill C, Owen C, Rudnicka A, Sadda SR, Taylor P, et al. Automated diabetic retinopathy image assessment softwares: Diagnostic accuracy and cost-effectiveness compared to human graders. Ophthalmologica. 2016;236 (Supplement 1):40-1. | Not a DTA study |
| European Society of R. Impact of artificial intelligence on radiology: a EuroAIM survey among members of the European Society of Radiology. Insights imaging. 2019;10(1):105 | Not a DTA study |
| Faes L, Wagner SK, Fu DJ, Liu X, Korot E, Ledsam JR, et al. Automated deep learning design for medical image classification by health-care professionals with no coding experience: a feasibility study. The Lancet Digital Health. 2019;1(5):e232-e42. | Internal validation only |
| Fatehi F, Jahedi F, Tay-Kearney ML, Kanagasingam Y. Teleophthalmology for the elderly population: A review of the literature. International Journal of Medical Informatics. 2020;136:104089. | Not a DTA study |
| Feng S, Zhuo Z, Pan D, Tian Q. CcNet: A cross-connected convolutional network for segmenting retinal vessels using multi-scale features. Neurocomputing. 2020;392:268-76. | Internal validation only |
| FengLi Y, Jing S, Annan L, Jun C, Cheng W, Jiang L. Image quality classification for DR screening using deep learning. Conf Proc IEEE Eng Med Biol Soc. 2017;2017:664-7. | Internal validation only |
| Fleming AD, Philip S, Goatman KA, Olson JA, Sharp PF. Automated microaneurysm detection using local contrast normalization and local vessel detection. IEEE Trans Med Imaging. 2006;25(9):1223-32. | Internal validation only |
| Fleming AD, Philip S, Goatman KA, Prescott GJ, Sharp PF, Olson JA. The evidence for automated grading in diabetic retinopathy screening. Curr Diabetes Rev. 2011;7(4):246-52. | Not a DTA study |
| Fleming AD, Philip S, Goatman KA, Williams GJ, Olson JA, Sharp PF. Automated detection of exudates for diabetic retinopathy screening. Phys Med Biol. 2007;52(24):7385-96. | Internal validation only |
| Francolini G, Desideri I, Stocchi G, Salvestrini V, Ciccone LP, Garlatti P, et al. Artificial Intelligence in radiotherapy: state of the art and future directions. Med Oncol. 2020;37(6):50. | Not a DTA study |
| Franklin SW, Rajan SE. An automated retinal imaging method for the early diagnosis of diabetic retinopathy. Technol Health Care. 2013;21(6):557-69. | Internal validation only |
| Ganesan K, Martis RJ, Acharya UR, Chua CK, Min LC, Ng EY, et al. Computer-aided diabetic retinopathy detection using trace transforms on digital fundus images. Med Biol Eng Comput. 2014;52(8):663-72. | Internal validation only |
| Garcia M, Lopez MI, Alvarez D, Hornero R. Assessment of four neural network based classifiers to automatically detect red lesions in retinal images. Med Eng Phys. 2010;32(10):1085-93. | Internal validation only |
| Garcia M, Sanchez CI, Lopez MI, Diez A, Hornero R. Automatic detection of red lesions in retinal images using a multilayer perceptron neural network. Conf Proc IEEE Eng Med Biol Soc. 2008;2008:5425-8. | Internal validation only |
| Garcia M, Sanchez CI, Poza J, Lopez MI, Hornero R. Detection of hard exudates in retinal images using a radial basis function classifier. Ann Biomed Eng. 2009;37(7):1448-63. | Internal validation only |
| Gargeya R, Leng T. Automated Identification of Diabetic Retinopathy Using Deep Learning. Ophthalmology. 2017;124(7):962-9. | Internal validation only |
| Garside K, Henderson R, Makarenko I, Masoller C. Topological data analysis of high resolution diabetic retinopathy images. PLoS ONE. 2019;14(5):e0217413. | Internal validation only |
| Ginestra JC, Giannini HM, Schweickert WD, Meadows L, Lynch MJ, Pavan K, et al. Clinician Perception of a Machine Learning-Based Early Warning System Designed to Predict Severe Sepsis and Septic Shock. Crit Care Med. 2019;47(11):1477-84. | Not a DTA study |
| Gonzalez-Gonzalo C, Liefers B, Vaidyanathan A, Van Zeeland H, Klaver CCW, Sanchez CI. Opening the "black box" of deep learning in automated screening of eye diseases. Investigative Ophthalmology and Visual Science Conference. 2019;60(9). | Not a DTA study |
| Gonzalez-Gonzalo C, Liefers B, van Ginneken B, Sanchez CI. Iterative Augmentation of Visual Evidence for Weakly-Supervised Lesion Localization in Deep Interpretability Frameworks: Application to Color Fundus Images. IEEE transactions on medical imaging. 2020;39(11):3499-511. | Aim |
| Gorges M, Sung J, Portales-Casamar E. Perceptions of expert and lay users on trust in the use of artificial intelligence for medical decision-making and risk prediction. Anesthesia and Analgesia. 2020;130 (5 Supplement 1):49-50. | Not a DTA study |
| Graham S, Depp C, Lee EE, Nebeker C, Tu X, Kim HC, et al. Artificial Intelligence for Mental Health and Mental Illnesses: an Overview. Curr Psychiatry Rep. 2019;21(11):116. | Not a DTA study |
| Gunasekeran DV, Ting DSW, Tan GSW, Wong TY. Artificial intelligence for diabetic retinopathy screening, prediction and management. Current opinion in ophthalmology. 2020;31(5):357-65. | Not a DTA study |
| Guo Y, Budak U, Sengur A. A novel retinal vessel detection approach based on multiple deep convolution neural networks. Comput Methods Programs Biomed. 2018;167:43-8. | Internal validation only |
| Halamka J, Cerrato P. An FP's guide to AI-enabled clinical decision support. Journal of Family Practice. 2019;68(9):486;8;90;92. | Not a DTA study |
| Hamilton SD. HTS automation study: Results from a 2001 survey of the current vs. desired state of HTS automation. JALA - Journal of the Association for Laboratory Automation. 2002;7(2):78-83. | Not a DTA study |
| Hansen M B, Tang H L, Wang S, Turk L A, Piermarocchi R, Speckauskas M, Hense HW, Leung I, Peto T. Automated detection of Diabetic Retinopathy in Three European Populations. J Clin Exp Ophthalmol 2016, 7:4; DOI: 10.4172/2155-9570.1000582. | Population |
| Harangi B, Hajdu A. Automatic exudate detection by fusing multiple active contours and regionwise classification. Comput Biol Med. 2014;54:156-71. | Internal validation only |
| Harangi B, Hajdu A. Detection of exudates in fundus images using a Markovian segmentation model. Conf Proc IEEE Eng Med Biol Soc. 2014;2014:130-3. | Internal validation only |
| Harangi B, Toth J, Baran A, Hajdu A. Automatic screening of fundus images using a combination of convolutional neural network and hand-crafted features. Conf Proc IEEE Eng Med Biol Soc. 2019;2019:2699-702. | Internal validation only |
| Hassan B, Hassan T, Li B, Ahmed R, Hassan O. Deep Ensemble Learning Based Objective Grading of Macular Edema by Extracting Clinically Significant Findings from Fused Retinal Imaging Modalities. Sensors (Basel). 2019;19(13):05. | Internal validation only |
| Hatanaka Y, Nakagawa T, Hayashi Y, Hara T, Fujita H. Improvement of automated detection method of hemorrhages in fundus images. Conf Proc IEEE Eng Med Biol Soc. 2008;2008:5429-32. | Internal validation only |
| Helmchen LA, Lehmann HP, Abramoff MD. Automated detection of retinal disease. Am J Manag Care. 2014;20(11 Spec No. 17):eSP48-52. | Not a DTA study |
| Hemanth DJ, Anitha J, Son LH, Mittal M. Diabetic Retinopathy Diagnosis from Retinal Images Using Modified Hopfield Neural Network. Journal of Medical Systems. 2018;42(12):247. | Internal validation only |
| Hsieh YT, Chuang LM, Jiang YD, Chang TJ, Yang CM, Yang CH, et al. Application of deep learning image assessment software VeriSee TM for diabetic retinopathy screening. J Formos Med Assoc. 2020;16:16. | Internal validation only |
| Hua C-H, Huynh-The T, Lee S. DRAN: Densely Reversed Attention based Convolutional Network for Diabetic Retinopathy Detection. Annual International Conference of the IEEE Engineering in Medicine and Biology Society IEEE Engineering in Medicine and Biology Society Annual International Conference. 2020;1992-5. | Internal validation only |
| Huang H, Ma H, Qian W. Automatic Parallel Detection of Neovascularization from Retinal Images Using Ensemble of Extreme Learning Machine<sup>.</sup>. Conf Proc IEEE Eng Med Biol Soc. 2019;2019:4712-6. | Internal validation only |
| Ibrahim S, Chowriappa P, Dua S, Acharya UR, Noronha K, Bhandary S, et al. Classification of diabetes maculopathy images using data-adaptive neuro-fuzzy inference classifier. Med Biol Eng Comput. 2015;53(12):1345-60. | Internal validation only |
| Imani E, Pourreza HR, Banaee T. Fully automated diabetic retinopathy screening using morphological component analysis. Comput Med Imaging Graph. 2015;43:78-88. | Internal validation only |
| Ipp E, Liljenquist DR. Reliability of an artificial intelligence (AI) diabetic retinopathy (DR) detection system. Diabetes. 2020;69(Supplement 1). | Not a full report |
| Islam MM, Yang HC, Poly TN, Jian WS, Jack Li YC. Deep learning algorithms for detection of diabetic retinopathy in retinal fundus photographs: A systematic review and meta-analysis. Comput Methods Programs Biomed. 2020;191:105320 | Not a DTA study |
| Jaafar HF, Nandi AK, Al-Nuaimy W. Automated detection of red lesions from digital colour fundus photographs. Conf Proc IEEE Eng Med Biol Soc. 2011;2011:6232-5. | Internal validation only |
| Jaafar HF, Nandi AK, Al-Nuaimy W. Decision support system for the detection and grading of hard exudates from color fundus photographs. J Biomed Opt. 2011;16(11):116001. | Internal validation only |
| Janaki SD, Geetha K. Enhanced CAE system for detection of exudates and diagnosis of diabetic retinopathy stages in fundus retinal images using soft computing techniques. Polish Journal of Medical Physics and Engineering. 2019;25(2):131-9. | Internal validation only |
| Jaya T, Dheeba J, Singh NA. Detection of Hard Exudates in Colour Fundus Images Using Fuzzy Support Vector Machine-Based Expert System. Journal of Digital Imaging. 2015;28(6):761-8. | Internal validation only |
| Jeba Derwin D, Tamil Selvi S, Jeba Singh O, Priestly Shan B. A novel automated system of discriminating Microaneurysms in fundus images. Biomedical Signal Processing and Control. 2020;58 (no pagination)(101839). | Internal validation only |
| Jelinek HF, Cree MJ, Leandro JJ, Soares JV, Cesar RM, Jr., Luckie A. Automated segmentation of retinal blood vessels and identification of proliferative diabetic retinopathy. J Opt Soc Am A Opt Image Sci Vis. 2007;24(5):1448-56. | Internal validation only |
| Jelinek HF, Rocha A, Carvalho T, Goldenstein S, Wainer J. Machine learning and pattern classification in identification of indigenous retinal pathology. Conf Proc IEEE Eng Med Biol Soc. 2011;2011:5951-4. | Internal validation only |
| Jheng YC, Chou YB, Kao CL, Yarmishyn AA, Hsu CC, Lin TC, et al. A Novelty Route for Smartphone-based Artificial Intelligence Approach to Ophthalmic Screening. J Chin Med Assoc. 2020;09:09. | Not a DTA study |
| Jiang J, Liu X, Liu L, Wang S, Long E, Yang H, et al. Predicting the progression of ophthalmic disease based on slit-lamp images using a deep temporal sequence network. PLoS ONE. 2018;13(7):e0201142. | Technology |
| Jiang J, Liu X, Zhang K, Long E, Wang L, Li W, et al. Automatic diagnosis of imbalanced ophthalmic images using a cost-sensitive deep convolutional neural network. Biomedical Engineering Online. 2017;16(1):132. | Target condition |
| Jiang Z, Yu Z, Feng S, Huang Z, Peng Y, Guo J, et al. A super-resolution method-based pipeline for fundus fluorescein angiography imaging. Biomedical Engineering Online. 2018;17(1):125. | Technology |
| Jiayi W, Jingmin X, Lai H, You J, Nanning Z. New hierarchical approach for microaneurysms detection with matched filter and machine learning. Conf Proc IEEE Eng Med Biol Soc. 2015;2015:4322-5. | Internal validation only |
| John S, Ram K, Sivaprakasam M, Raman R. Assessment of Computer-Assisted Screening Technology for Diabetic Retinopathy Screening in India - Preliminary Results and Recommendations from a Pilot Study. Studies in Health Technology & Informatics. 2016;231:74-81. | Internal validation only |
| Jonmarker O, Strand F, Brandberg Y, Lindholm P. The future of breast cancer screening: what do participants in a breast cancer screening program think about automation using artificial intelligence? Acta Radiol Open. 2019;8(12):2058460119880315. | Not a DTA study |
| Joonyoung S, Boreom L. Development of automatic retinal vessel segmentation method in fundus images via convolutional neural networks. Conf Proc IEEE Eng Med Biol Soc. 2017;2017:681-4. | Internal validation only |
| Joshi S, Karule PT. Mathematical morphology for microaneurysm detection in fundus images. European Journal of Ophthalmology. 2019:1120672119843021. | Internal validation only |
| Jungmann F, Jorg T, Hahn F, Pinto Dos Santos D, Jungmann SM, Duber C, et al. Attitudes Toward Artificial Intelligence Among Radiologists, IT Specialists, and Industry. Acad Radiol. 2020;12:12 | Not a DTA study |
| Kande GB, Subbaiah PV, Savithri TS. Unsupervised fuzzy based vessel segmentation in pathological digital fundus images. Journal of Medical Systems. 2010;34(5):849-58. | Internal validation only |
| Kandemir M, Hamprecht FA. Computer-aided diagnosis from weak supervision: a benchmarking study. Comput Med Imaging Graph. 2015;42:44-50. | Internal validation only |
| Kapetanakis VV, Rudnicka AR, Liew G, Owen CG, Lee A, Louw V, et al. A study of whether automated Diabetic Retinopathy Image Assessment could replace manual grading steps in the English National Screening Programme. J Med Screen. 2015;22(3):112-8. | Not a DTA study |
| Kapoor R, Whigham BT, Al-Aswad LA. Artificial Intelligence and Optical Coherence Tomography Imaging. Asia Pac J Ophthalmol (Phila). 2019;8(2):187-94. | Not a DTA study |
| Kapoor R, Whigham BT, Al-Aswad LA. The Role of Artificial Intelligence in the Diagnosis and Management of Glaucoma. Current Ophthalmology Reports. 2019;7(2):136-42. | Not a DTA study |
| Karnowski TP, Aykac D, Giancardo L, Li Y, Nichols T, Tobin KW, Jr., et al. Automatic detection of retina disease: robustness to image quality and localization of anatomy structure. Conf Proc IEEE Eng Med Biol Soc. 2011;2011:5959-64. | Internal validation only |
| Karperien A, Jelinek HF, Leandro JJ, Soares JV, Cesar RM, Jr., Luckie A. Automated detection of proliferative retinopathy in clinical practice. Clinical Ophthalmology. 2008;2(1):109-22. | Internal validation only |
| Karthikeyan R, Alli P. Feature Selection and Parameters Optimization of Support Vector Machines Based on Hybrid Glowworm Swarm Optimization for Classification of Diabetic Retinopathy. Journal of Medical Systems. 2018;42(10):195. | Internal validation only |
| Karthikeyan S, Sanjay Kumar P, Madhusudan RJ, Sundaramoorthy SK, Krishnan Namboori PK. Detection of multi-class retinal diseases using artificial intelligence: An expeditious learning using deep CNn with minimal data. Biomedical and Pharmacology Journal. 2019;12(3):1577-86. | Internal validation only |
| Kauppi T, Kamarainen JK, Lensu L, Kalesnykiene V, Sorri I, Uusitalo H, et al. Constructing benchmark databases and protocols for medical image analysis: diabetic retinopathy. Comput. 2013;2013:368514. | Not a DTA study |
| Kavitha G, Ramakrishnan S. Abnormality detection in retinal images using ant colony optimization and artificial neural networks - biomed 2010. Biomed Sci Instrum. 2010;46:331-6. | Internal validation only |
| Keane PA. Artificial intelligence: The algorithmic solution to retinal healthcare. Investigative Ophthalmology and Visual Science Conference. 2019;60(9). | Not a DTA study |
| Keel S, Lee PY, Scheetz J, Li Z, Kotowicz MA, MacIsaac RJ, et al. Feasibility and patient acceptability of a novel artificial intelligence-based screening model for diabetic retinopathy at endocrinology outpatient services: a pilot study. Sci. 2018;8(1):4330. | Not a DTA study |
| Keel S, Wu J, Lee PY, Scheetz J, He M. Visualizing Deep Learning Models for the Detection of Referable Diabetic Retinopathy and Glaucoma. JAMA Ophthalmology. 2019;137(3):288-92. | Aim |
| Kermany DS, Goldbaum M, Cai W, Valentim CCS, Liang H, Baxter SL, et al. Identifying Medical Diagnoses and Treatable Diseases by Image-Based Deep Learning. Cell. 2018;172(5):1122-31.e9. | Technology |
| Kesim C, Tas AY, Karslioglu MZ, Ozkaya A, Gokgur E, Cakin I, et al. Validation results of a deep learning algorithm for detection of diabetic retinopathy with lesion localization from retinal fundus photographs. Investigative Ophthalmology and Visual Science. 2020;61(7). | Not a full report |
| Keskinbora K, Guven F. Artificial intelligence and ophthalmology. Turkish Journal of Ophthalmology. 2020;50(1):37-43. | Not a DTA study |
| Khojasteh P, Aliahmad B, Kumar DK. Fundus images analysis using deep features for detection of exudates, hemorrhages and microaneurysms. BMC ophthalmol. 2018;18(1):288. | Internal validation only |
| Khojasteh P, Passos Junior LA, Carvalho T, Rezende E, Aliahmad B, Papa JP, et al. Exudate detection in fundus images using deeply-learnable features. Comput Biol Med. 2019;104:62-9. | Internal validation only |
| Khomri B, Christodoulidis A, Djerou L, Babahenini MC, Cheriet F. Particle swarm optimization method for small retinal vessels detection on multiresolution fundus images. J Biomed Opt. 2018;23(5):1-13. | Internal validation only |
| Koch M. Artificial Intelligence Is Becoming Natural. Cell. 2018;173(3):531-3. | Not a DTA study |
| Koh DM. Attitudes and perception of artificial intelligence and machine learning in oncological imaging. Cancer Imaging Conference: 19th Meeting and Annual of the International Cancer Imaging Society Italy. 2019;19(Supplement 1). | Not a DTA study |
| Kose C, Sevik U, Ikibas C, Erdol H. Simple methods for segmentation and measurement of diabetic retinopathy lesions in retinal fundus images. Comput Methods Programs Biomed. 2012;107(2):274-93. | Internal validation only |
| Krach S, Hegel F, Wrede B, Sagerer G, Binkofski F, Kircher T. Can machines think? Interaction and perspective taking with robots investigated via fMRI. PLoS ONE. 2008;3(7):e2597. | Not a DTA study |
| Krishnan MMR, Laude A. An integrated diabetic retinopathy index for the diagnosis of retinopathy using digital fundus image features. Journal of Medical Imaging and Health Informatics. 2013;3(2):306-13. | Internal validation only |
| Kusakunniran W, Wu Q, Ritthipravat P, Zhang J. Hard exudates segmentation based on learned initial seeds and iterative graph cut. Comput Methods Programs Biomed. 2018;158:173-83. | Internal validation only |
| Lahmiri S, Boukadoum M. Automated detection of circinate exudates in retina digital images using empirical mode decomposition and the entropy and uniformity of the intrinsic mode functions. Biomed Tech (Berl). 2014;59(4):357-66. | Internal validation only |
| Lahmiri S. Hybrid deep learning convolutional neural networks and optimal nonlinear support vector machine to detect presence of hemorrhage in retina. Biomedical Signal Processing and Control. 2020;60 (no pagination)(101978). | Internal validation only |
| Lam BY, Yan H. A novel vessel segmentation algorithm for pathological retina images based on the divergence of vector fields. IEEE Trans Med Imaging. 2008;27(2):237-46. | Internal validation only |
| Lam C, Yi D, Guo M, Lindsey T. Automated Detection of Diabetic Retinopathy using Deep Learning. AMIA Summits Transl Sci Proc. 2018;2017:147-55. | Internal validation only |
| Lamard M, Matta SCZ, Rottier J-B, Cochener B, Massin P, Quellec G. Evaluation of an automatic screening system for detecting retinal pathologies. Investigative Ophthalmology and Visual Science. 2020;61(7). | Not a full report |
| Larson DB, Magnus DC, Lungren MP, Shah NH, Langlotz CP. Ethics of Using and Sharing Clinical Imaging Data for Artificial Intelligence: A Proposed Framework. Radiology. 2020;295(3):675-82. | Not a DTA study |
| Leibig C, Allken V, Ayhan MS, Berens P, Wahl S. Leveraging uncertainty information from deep neural networks for disease detection. Sci. 2017;7(1):17816. | Internal validation only |
| Lemke HU. Machine intelligence and CARS. International Journal of Computer Assisted Radiology and Surgery. 2018;13 (Supplement 1):S125-S6. | Not a DTA study |
| Li F, Liu Z, Chen H, Jiang M, Zhang X, Wu Z. Automatic Detection of Diabetic Retinopathy in Retinal Fundus Photographs Based on Deep Learning Algorithm. Transl. 2019;8(6):4. | Internal validation only |
| Li Q, Fan S, Chen C. An Intelligent Segmentation and Diagnosis Method for Diabetic Retinopathy Based on Improved U-NET Network. Journal of Medical Systems. 2019;43(9):304. | Internal validation only |
| Li Z, Guo C, Nie D, Lin D, Yi Z, Chen C, et al. Development and evaluation of a deep learning system for screening retinal hemorrhage based on ultra-wide-field fundus images. Translational Vision Science and Technology. 2020;9 (2) (no pagination)(3). | Technology |
| Liew CJ, Krishnaswamy P, Cheng LT, Tan CH, Poh AC, Lim TC. Artificial Intelligence and Radiology in Singapore: Championing a New Age of Augmented Imaging for Unsurpassed Patient Care. Ann Acad Med Singapore. 2019;48(1):16-24. | Not a DTA study |
| Liew G, Egan CA, Rudnicka A, Owen C, Kapetan V, Sim DA, et al. Evaluation of automated software grading of diabetic retinopathy and comparison with manual image grading-an accuracy and cost effectiveness study. Investigative Ophthalmology and Visual Science. 2014;55 (13):2293 | Not a DTA study |
| Lim J BM, Ramachandra C, Bhat S, Solanki K, Sadda S. Artificial Intelligence Screening for Diabetic Retinopathy: Analysis from a Pivotal Multi-Center Prospective Clinical Trial. ARVO Imaging in the Eye Conference 2019; Vancouver, BC, Canada: ARVO; 2019. | Not a full report |
| Lin GM, Chen MJ, Yeh CH, Lin YY, Kuo HY, Lin MH, et al. Transforming Retinal Photographs to Entropy Images in Deep Learning to Improve Automated Detection for Diabetic Retinopathy. Journal of ophthalmology. 2018;2018:2159702. | Internal validation only |
| Lin H, Li R, Liu Z, Chen J, Yang Y, Chen H, et al. Diagnostic Efficacy and Therapeutic Decision-making Capacity of an Artificial Intelligence Platform for Childhood Cataracts in Eye Clinics: A Multicentre Randomized Controlled Trial. EClinicalMedicine. 2019;9:52-9. | Not a DTA study |
| Liu YP, Li Z, Xu C, Li J, Liang R. Referable diabetic retinopathy identification from eye fundus images with weighted path for convolutional neural network. Artificial Intelligence in Medicine. 2019;99:101694. | Internal validation only |
| Liu Z, Yao Z, Cao Y, Wu J. Computerized diagnosis of fundus vascular structure based on predictions of diabetic retinopathy grade and risk of macular edema. Journal of Medical Imaging and Health Informatics. 2019;9(5):884-92. | Internal validation only |
| Long S, Huang X, Chen Z, Pardhan S, Zheng D. Automatic Detection of Hard Exudates in Color Retinal Images Using Dynamic Threshold and SVM Classification: Algorithm Development and Evaluation. Biomed Res Int. 2019;2019:3926930. | Outcomes |
| Lu, Z E, Chen, Ye C, Ooms A, Szirth B, Khouri A S. Accuracy of automated retinal software for diabetic retinopathy detection in type I diabetics vs human graders. Investigative Ophthalmology and Visual Science. 2020;61(7). | Not a full report |
| Lyford T, Sheppard J. Diabetic Eye Disease: Advancements in Technology, Detection, and Access to Care. Sr Care Pharm. 2020;35(6):266-72. | Not a DTA study |
| Mansour RF. Deep-learning-based automatic computer-aided diagnosis system for diabetic retinopathy. Biomedical Engineering Letters. 2018;8(1):41-57. | Internal validation only |
| Mathenge WC. Artificial intelligence for diabetic retinopathy screening in Africa. The Lancet Digital Health. 2019;1(1):e6-e7. | Not a DTA study |
| Meyer AND, Giardina TD, Spitzmueller C, Shahid U, Scott TMT, Singh H. Patient Perspectives on the Usefulness of an Artificial Intelligence-Assisted Symptom Checker: CrossSectional Survey Study. J Med Internet Res. 2020;22(1):e14679. | Not a DTA study |
| Meza-Kubo V, Morán AL, Carrillo I, Galindo G, García-Canseco E. Assessing the user experience of older adults using a neural network trained to recognize emotions from brain signals. Journal of Biomedical Informatics. 2016;62:202-9. | Not a DTA study |
| Mokhashi N, Grachevskaya J, Cheng L, Yu D, Henderer J. A comparison of artificial intelligence and human diabetic retinal image interpretation in an urban health system. Investigative Ophthalmology and Visual Science. 2020;61(7). | Not a full report |
| Mookiah MR, Acharya UR, Chandran V, Martis RJ, Tan JH, Koh JE, et al. Application of higher-order spectra for automated grading of diabetic maculopathy. Med Biol Eng Comput. 2015;53(12):1319-31. | Internal validation only |
| Mumtaz R, Hussain M, Sarwar S, Khan K, Mumtaz S, Mumtaz M. Automatic detection of retinal hemorrhages by exploiting image processing techniques for screening retinal diseases in diabetic patients. International Journal of Diabetes in Developing Countries. 2018;38(1):80-7. | Internal validation only |
| Murugeswari S, Sukanesh R. Examinations on diffuse diabetic macular oedema using neural networks. Journal of Medical Imaging and Health Informatics. 2016;6(8):2019-23. | Internal validation only |
| Murugeswari S, Sukanesh R. Investigations of severity level measurements for diabetic macular oedema using machine learning algorithms. Ir J Med Sci. 2017;186(4):929-38. | Internal validation only |
| Nadarzynski T, Miles O, Cowie A, Ridge D. Acceptability of artificial intelligence (AI)-led chatbot services in healthcare: A mixed-methods study. DIGITAL HEALTH. 2019;5:2055207619871808. | Not a DTA study |
| Nagendran M, Chen Y, Lovejoy CA, Gordon AC, Komorowski M, Harvey H, et al. Artificial intelligence versus clinicians: Systematic review of design, reporting standards, and claims of deep learning studies in medical imaging. The BMJ. 2020;368 (no pagination)(m689). | Not a DTA study |
| Naqvi SA, Zafar MF, Haq I. Referral system for hard exudates in eye fundus. Comput Biol Med. 2015;64:217-35. | Internal validation only |
| Naqvi SAG, Zafar HMF, Ul Haq I. Automated System for Referral of Cotton-Wool Spots. Curr Diabetes Rev. 2018;14(2):168-74. | Internal validation only |
| Narasimha-Iyer H, Can A, Roysam B, Stewart CV, Tanenbaum HL, Majerovics A, et al. Robust detection and classification of longitudinal changes in color retinal fundus images for monitoring diabetic retinopathy. IEEE Trans Biomed Eng. 2006;53(6):1084-98. | Aim |
| Narasimha-Iyer H, Can A, Roysam B, Tanenbaum HL, Majerovics A. Integrated analysis of vascular and nonvascular changes from color retinal fundus image sequences. IEEE Trans Biomed Eng. 2007;54(8):1436-45. | Aim |
| Nayak J, Bhat PS, Acharya R, Lim CM, Kagathi M. Automated identification of diabetic retinopathy stages using digital fundus images. Journal of Medical Systems. 2008;32(2):107-15. | Internal validation only |
| Nayak J, Bhat PS, Acharya UR. Automatic identification of diabetic maculopathy stages using fundus images. J Med Eng Technol. 2009;33(2):119-29. | Internal validation only |
| Nazir T, Irtaza A, Shabbir Z, Javed A, Akram U, Mahmood MT. Diabetic retinopathy detection through novel tetragonal local octa patterns and extreme learning machines. Artificial Intelligence in Medicine. 2019;99:101695. | Internal validation only |
| Nguyen PA, Li YC. Artificial Intelligence in Clinical Implications. Computer Methods and Programs in Biomedicine. 2018;166:A1. | Not a DTA study |
| Nidhi MT, Gunaseelan K. Efficient ranking of diabetic retinopathy and glaucoma using echo state neural network and radial basis function (RBF). Journal of Medical Imaging and Health Informatics. 2016;6(3):869-74. | Internal validation only |
| Nielsen KB, Lautrup ML, Andersen JKH, Savarimuthu TR, Grauslund J. Deep LearningBased Algorithms in Screening of Diabetic Retinopathy: A Systematic Review of Diagnostic Performance. Ophthalmol Retina. 2019;3(4):294-304. | Not a DTA study |
| Niemeijer M, van Ginneken B, Russell SR, Suttorp-Schulten MS, Abramoff MD. Automated detection and differentiation of drusen, exudates, and cotton-wool spots in digital color fundus photographs for diabetic retinopathy diagnosis. Invest Ophthalmol Vis Sci. 2007;48(5):2260-7. | Internal validation only |
| Nørgaard MF, Grauslund J. Automated Screening for Diabetic Retinopathy - A Systematic Review. Ophthalmic Res. 2018;60(1):9-17. | Not a DTA study |
| Noronha K, Acharya UR, Nayak KP, Kamath S, Bhandary SV. Decision support system for diabetic retinopathy using discrete wavelet transform. Proc Inst Mech Eng [H]. 2013;227(3):251-61. | Internal validation only |
| O'Connor AM, Tsafnat G, Thomas J, Glasziou P, Gilbert SB, Hutton B. A question of trust: can we build an evidence base to gain trust in systematic review automation technologies? Syst. 2019;8(1):143. | Not a DTA study |
| Ogunyemi O, Moran E, Daskivich LP, George S, Teklehaimanot S, Ilapakurthi R, et al. Autonomy versus automation: Perceptions of nonmydriatic camera choice for teleretinal screening in an urban safety net clinic. Telemedicine and e-Health. 2013;19(8):591-6. | Aim |
| Olson J, Sharp P, Goatman K, Prescott G, Scotland G, Fleming A, et al. Improving the economic value of photographic screening for optical coherence tomography-detectable macular oedema: a prospective, multicentre, UK study. Health Technol Assess. 2013;17(51):1-142. | Not a DTA study |
| Ooi SKG, Makmur A, Soon AYQ, Fook-Chong S, Liew C, Sia SY, et al. Attitudes toward artificial intelligence in radiology with learner needs assessment within radiology residency programmes: a national multi-programme survey. Singapore Med J. 2019;04:04 | Not a DTA study |
| Ooms A, Caterfino A, Prasad N, Khouri P, Wilson L, Szirth B. Robotics and artificial intelligence in the management of vision threatening disease. Investigative Ophthalmology and Visual Science Conference. 2019;60(9). | Not a DTA study |
| Ordonez PF, Cepeda CM, Garrido J, Chakravarty S. Classification of images based on small local features: a case applied to microaneurysms in fundus retina images. Journal of Medical Imaging. 2017;4(4):041309. | Outcomes |
| Osareh A, Mirmehdi M, Thomas B, Markham R. Automated identification of diabetic retinal exudates in digital colour images. British Journal of Ophthalmology. 2003;87(10):1220-3. | Internal validation only |
| Osareh A, Shadgar B, Markham R. A computational-intelligence-based approach for detection of exudates in diabetic retinopathy images. IEEE Trans Inf Technol Biomed. 2009;13(4):535-45. | Internal validation only |
| Padhy SK, Takkar B, Chawla R, Kumar A. Artificial intelligence in diabetic retinopathy: A natural step to the future. Indian J Ophthalmol. 2019;67(7):1004-9. | Not a DTA study |
| Palmisciano P, Jamjoom AAB, Taylor D, Stoyanov D, Marcus HJ. Attitudes of Patients and Their Relatives Toward Artificial Intelligence in Neurosurgery. World Neurosurg. 2020;138:e627-e33. | Not a DTA study |
| Pan X, Jin K, Cao J, Liu Z, Wu J, You K, et al. Multi-label classification of retinal lesions in diabetic retinopathy for automatic analysis of fundus fluorescein angiography based on deep learning. Graefe's Archive for Clinical and Experimental Ophthalmology. 2020;258(4):779-85. | Technology |
| Parekh NU, Bhaskaranand M, Ramachandra C, Bhat S, Solanki K. Explaining an artificial intelligence (AI) system for diabetic retinopathy (DR) screening in primary care. Diabetes Conference: 79th Scientific Sessions of the American Diabetes Association, ADA. 2019;68(Supplement 1). | Not a DTA study |
| Parthasarathy D R, Savoy F, Sosale B, Sosale A, Narayana S, Sharma U et al. Performance of screening algorithms for Referable Diabetic Retinopathy (RDR) and Sight Threatening Diabetic Retinopathy (STDR) on a non-mydriatic portable smartphone-based fundus camera. Investigative Ophthalmology and Visual Science. 2020;61(7). | Not a full report |
| Patel JL, Goyal RK. Applications of artificial neural networks in medical science. Current Clinical Pharmacology. 2007;2(3):217-26. | Not a DTA study |
| Paul PG, Raman R, Rani PK, Deshmukh H, Sharma T. Patient satisfaction levels during teleophthalmology consultation in rural South India. Telemed J E Health. 2006;12(5):571-8. | Not a DTA study |
| Pedrosa M, Silva JM, Matos S, Costa C. SCREEN-DR - Software Architecture for the Diabetic Retinopathy Screening. Studies in Health Technology & Informatics. 2018;247:396-400. | Not a DTA study |
| Pedrosa M, Silva JM, Silva JF, Matos S, Costa C. SCREEN-DR: Collaborative platform for diabetic retinopathy. International Journal of Medical Informatics. 2018;120:137-46. | Not a DTA study |
| Philip S, Lee N, Black M, Sharp P, Olson J. Impact of introducing automated grading into the Scottish national diabetic retinopathy screening programme. Diabetic Medicine. 2017;34 (Supplement 1):172. | Not a full report |
| Pires R, Avila S, Jelinek HF, Wainer J, Valle E, Rocha A. Automatic Diabetic Retinopathy detection using BossaNova representation. Conf Proc IEEE Eng Med Biol Soc. 2014;2014:146-9. | Internal validation only |
| Playout C, Duval R, Cheriet F. A Novel Weakly Supervised Multitask Architecture for Retinal Lesions Segmentation on Fundus Images. IEEE Trans Med Imaging. 2019;38(10):2434-44. | Internal validation only |
| Poly TN, Islam MM, Yang HC, Nguyen PA, Wu CC, Li YJ. Artificial Intelligence in Diabetic Retinopathy: Insights from a Meta-Analysis of Deep Learning. Studies in Health Technology & Informatics. 2019;264:1556-7. | Not a DTA study |
| Porwal P, Pachade S, Kokare M, Giancardo L, Meriaudeau F. Retinal image analysis for disease screening through local tetra patterns. Comput Biol Med. 2018;102:200-10. | Internal validation only |
| Prakash NB, Hemalakshmi GR, Stella Inba Mary M. Automated grading of diabetic retinopathy stages in fundus images using SVM classifer. Journal of Chemical and Pharmaceutical Research. 2016;8(1):537-41. | Internal validation only |
| Prentasic P, Loncaric S. Detection of exudates in fundus photographs using deep neural networks and anatomical landmark detection fusion. Comput Methods Programs Biomed. 2016;137:281-92. | Internal validation only |
| Prentasic P, Loncaric S. Weighted ensemble based automatic detection of exudates in fundus photographs. Conf Proc IEEE Eng Med Biol Soc. 2014;2014:138-41. | Internal validation only |
| Prescott G, Sharp P, Goatman K, Scotland G, Fleming A, Philip S, et al. Improving the costeffectiveness of photographic screening for diabetic macular oedema: a prospective, multi-centre, UK study. British Journal of Ophthalmology. 2014;98(8):1042-9. | Not a DTA study |
| Punniyamoorthy U, Pushpam I. Remote examination of exudates-impact of macular oedema. Healthc. 2018;5(4):118-23. | Internal validation only |
| Quellec G, Charriere K, Boudi Y, Cochener B, Lamard M. Deep image mining for diabetic retinopathy screening. Med Image Anal. 2017;39:178-93. | Internal validation only |
| Quellec G, Lamard M, Abramoff MD, Decenciere E, Lay B, Erginay A, et al. A multiple-instance learning framework for diabetic retinopathy screening. Med Image Anal. 2012;16(6):1228-40. | Internal validation only |
| Quellec G, Lamard M, Cazuguel G, Bekri L, Daccache W, Roux C, et al. Automated assessment of diabetic retinopathy severity using content-based image retrieval in multimodal fundus photographs. Invest Ophthalmol Vis Sci. 2011;52(11):8342-8. | Internal validation only |
| Quellec G, Lamard M, Conze PH, Massin P, Cochener B. Automatic detection of rare pathologies in fundus photographs using few-shot learning. Med Image Anal. 2020;61:101660. | Internal validation only |
| Quellec G, Lamard M, Erginay A, Chabouis A, Massin P, Cochener B, et al. Automatic detection of referral patients due to retinal pathologies through data mining. Med Image Anal. 2016;29:47-64. | Target condition |
| Quellec G, Lamard M, Josselin PM, Cazuguel G, Cochener B, Roux C. Detection of lesions in retina photographs based on the wavelet transform. Conf Proc IEEE Eng Med Biol Soc. 2006;2006:2618-21. | Internal validation only |
| Quellec G, Lamard M, Josselin PM, Cazuguel G, Cochener B, Roux C. Optimal wavelet transform for the detection of microaneurysms in retina photographs. IEEE Trans Med Imaging. 2008;27(9):1230-41. | Internal validation only |
| Quellec G, Russell SR, Abramoff MD. Optimal filter framework for automated, instantaneous detection of lesions in retinal images. IEEE Trans Med Imaging. 2011;30(2):523-33. | Internal validation only |
| Rahimy E. Deep learning applications in ophthalmology. Current Opinion in Ophthalmology. 2018;29(3):254-60. | Not a DTA study |
| Rajalakshmi R. The impact of artificial intelligence in screening for diabetic retinopathy in India. Eye. 2020;34(3):420-1. | Not a DTA study |
| Rajesh IS, Arikerie BM, Reshmi BM. A review on automatic identification of fovea in retinal fundus images. International Journal of Medical Engineering and Informatics. 2020;12(2):169-79. | Not a DTA study |
| Raju M, Pagidimarri V, Barreto R, Kadam A, Kasivajjala V, Aswath A. Development of a Deep Learning Algorithm for Automatic Diagnosis of Diabetic Retinopathy. Studies in Health Technology & Informatics. 2017;245:559-63. | Internal validation only |
| Randive SN, Senapati RK, Rahulkar AD. A self-adaptive optimisation for diabetic retinopathy detection with neural classification. International Journal of Nano and Biomaterials. 2019;8(3-4):204-27. | Internal validation only |
| Reeves A. ES08.07 System Approach to Screening Management. Journal of Thoracic Oncology. 2019;14 (10 Supplement):S33-S4. | Not a DTA study |
| Ren F, Cao P, Li W, Zhao D, Zaiane O. Ensemble based adaptive over-sampling method for imbalanced data learning in computer aided detection of microaneurysm. Comput Med Imaging Graph. 2017;55:54-67. | Internal validation only |
| Ren F, Cao P, Zhao D, Wan C. Diabetic macular edema grading in retinal images using vector quantization and semi-supervised learning. Technol Health Care. 2018;26(S1):389-97. | Internal validation only |
| Reza AW, Eswaran C. A decision support system for automatic screening of non-proliferative diabetic retinopathy. Journal of Medical Systems. 2011;35(1):17-24. | Internal validation only |
| Riaz H, Park J, Choi H, Kim H, Kim J. Deep and Densely Connected Networks for Classification of Diabetic Retinopathy. Diagnostics. 2020;10(1):02. | Internal validation only |
| Rocha A, Carvalho T, Jelinek HF, Goldenstein S, Wainer J. Points of interest and visual dictionaries for automatic retinal lesion detection. IEEE Trans Biomed Eng. 2012;59(8):2244-53. | Internal validation only |
| Roychowdhury S, Koozekanani DD, Parhi KK. Automated detection of neovascularization for proliferative diabetic retinopathy screening. Conf Proc IEEE Eng Med Biol Soc. 2016;2016:1300-3. | Internal validation only |
| Roychowdhury S, Koozekanani DD, Parhi KK. DREAM: diabetic retinopathy analysis using machine learning. IEEE j. 2014;18(5):1717-28. | Internal validation only |
| Ruamviboonsuk P, Cheung CY, Zhang X, Raman R, Park SJ, Ting DSW. Artificial Intelligence in Ophthalmology: Evolutions in Asia. Asia Pac J Ophthalmol (Phila). 2020;9(2):78-84. | Not a DTA study |
| S KS, P A. A Machine Learning Ensemble Classifier for Early Prediction of Diabetic Retinopathy. Journal of Medical Systems. 2017;41(12):201. | Internal validation only |
| Saha SK, Fernando B, Cuadros J, Xiao D, Kanagasingam Y. Automated Quality Assessment of Colour Fundus Images for Diabetic Retinopathy Screening in Telemedicine. Journal of Digital Imaging. 2018;31(6):869-78. | Aim |
| Saha SK, Xiao D, Kanagasingam Y. A Novel Method for Correcting Non-uniform/Poor Illumination of Color Fundus Photographs. Journal of Digital Imaging. 2018;31(4):553-61. | Aim |
| Sahlsten J, Jaskari J, Kivinen J, Turunen L, Jaanio E, Hietala K, et al. Deep Learning Fundus Image Analysis for Diabetic Retinopathy and Macular Edema Grading. Sci. 2019;9(1):10750. | Internal validation only |
| Saleh E, Blaszczynski J, Moreno A, Valls A, Romero-Aroca P, de la Riva-Fernandez S, et al. Learning ensemble classifiers for diabetic retinopathy assessment. Artificial Intelligence in Medicine. 2018;85:50-63. | Internal validation only |
| Sanchez CI, Garcia M, Mayo A, Lopez MI, Hornero R. Retinal image analysis based on mixture models to detect hard exudates. Med Image Anal. 2009;13(4):650-8. | Internal validation only |
| Sanchez CI, Niemeijer M, Abramoff MD, van Ginneken B. Active learning for an efficient training strategy of computer-aided diagnosis systems: application to diabetic retinopathy screening. Med Image Comput Comput Assist Interv Int Conf Med Image Comput Comput Assist Interv. 2010;13(Pt 3):603-10. | Internal validation only |
| Sánchez CI, Niemeijer M, Dumitrescu AV, Suttorp-Schulten MS, Abràmoff MD, van Ginneken B. Evaluation of a computer-aided diagnosis system for diabetic retinopathy screening on public data. Invest Ophthalmol Vis Sci. 2011 Jul 1;52(7):4866-71. doi: 10.1167/iovs.10-6633. PMID: 21527381. | Technology |
| Sangeethaa SN, Uma Maheswari P. An Intelligent Model for Blood Vessel Segmentation in Diagnosing DR Using CNN. Journal of Medical Systems. 2018;42(10):175. | Internal validation only |
| Santhi D, Manimegalai D, Parvathi S, Karkuzhali S. Segmentation and classification of bright lesions to diagnose diabetic retinopathy in retinal images. Biomed Tech (Berl). 2016;61(4):443-53. | Internal validation only |
| Savoy M. IDx-DR for Diabetic Retinopathy Screening. Am Fam Physician. 2020;101(5):307-8. | Not a DTA study |
| Sayres R, Taly A, Rahimy E, Blumer K, Coz D, Hammel N, et al. Using a Deep Learning Algorithm and Integrated Gradients Explanation to Assist Grading for Diabetic Retinopathy. Ophthalmology. 2019;126(4):552-64. | Aim |
| Scanlon PH. Update on Screening for Sight-Threatening Diabetic Retinopathy. Ophthalmic Res. 2019;62(4):218-24. | Not a DTA study |
| Scotland GS, McNamee P, Fleming AD, Goatman KA, Philip S, Prescott GJ, et al. Costs and consequences of automated algorithms versus manual grading for the detection of referable diabetic retinopathy. British Journal of Ophthalmology. 2010;94(6):712-9 | Not a DTA study |
| Scotland GS, McNamee P, Philip S, Fleming AD, Goatman KA, Prescott GJ, et al. Costeffectiveness of implementing automated grading within the national screening programme for diabetic retinopathy in Scotland. British Journal of Ophthalmology. 2007;91(11):1518-23. | Not a DTA study |
| Scott IA, Cook D, Coiera EW, Richards B. Machine learning in clinical practice: prospects and pitfalls. Medical Journal of Australia. 2019;211(5):203-5. | Not a DTA study |
| Shaban M, Ogur Z, Mahmoud A, Switala A, Shalaby A, Abu Khalifeh H, et al. A convolutional neural network for the screening and staging of diabetic retinopathy. PLoS ONE. 2020;15(6):e0233514. | Internal validation only |
| Shaban-Nejad A, Michalowski M, Buckeridge DL. Health intelligence: how artificial intelligence transforms population and personalized health. npj digit. 2018;1:53. | Not a DTA study |
| Shah P, Mishra DK, Shanmugam MP, Doshi B, Jayaraj H, Ramanjulu R. Validation of Deep Convolutional Neural Network-based algorithm for detection of diabetic retinopathy - Artificial intelligence versus clinician for screening. Indian J Ophthalmol. 2020;68(2):398-405. | Internal validation only |
| Sharma P, Nirmala SR, Sarma KK. Classification of retinal images using image processing techniques. Journal of Medical Imaging and Health Informatics. 2013;3(3):341-6. | Internal validation only |
| Sharma S, Maheshwari S, Shukla A. An intelligible deep convolution neural network based approach for classification of diabetic retinopathy. Bio-Algorithms and Med-Systems. 2018;14 (2) (no pagination)(20180011). | Internal validation only |
| Shuang Y, Di X, Kanagasingam Y. Automatic detection of neovascularization on optic disk region with feature extraction and support vector machine. Conf Proc IEEE Eng Med Biol Soc. 2016;2016:1324-7. | Internal validation only |
| Shuang Y, Di X, Kanagasingam Y. Exudate detection for diabetic retinopathy with convolutional neural networks. Conf Proc IEEE Eng Med Biol Soc. 2017;2017:1744-7. | Internal validation only |
| Sim D. Historical perspective of diabetic retinopathy screening in the united kingdom - Where do we go from here? West Indian Medical Journal. 2018;67 (Supplement 1):19. | Not a DTA study |
| Simoes PW, Dos Passos MG, Amaral LL, Garcia D, Vicente RB, de Abreu LLT, et al. MetaAnalysis of the Sensitivity of Decision Support Systems in Diagnosing Diabetic Retinopathy. Studies in Health Technology & Informatics. 2019;264:878-82. | Not a DTA study |
| Singh RK, Gorantla R. DMENet: Diabetic Macular Edema diagnosis using Hierarchical Ensemble of CNNs. PLoS ONE. 2020;15(2):e0220677. | Internal validation only |
| Sinthanayothin C, Boyce JF, Williamson TH, Cook HL, Mensah E, Lal S, et al. Automated detection of diabetic retinopathy on digital fundus images. Diabetic Medicine. 2002;19(2):105-12. | Outcomes |
| Sivaprasad S, Raman R, Conroy D, Mohan t, Wittenberg R, Rajalakshmi R, et al. The ORNATE India Project: United Kingdom-India Research Collaboration to tackle visual impairment due to diabetic retinopathy. Eye. 2020;34(7):1279-86. | Not a DTA study |
| Soares F, Dutra-Medeiros M, Monteiro-Soares M, Rego S. Screening for Diabetic Retinopathy Using an Automated Diagnostic System Based on Deep Learning: Diagnostic Accuracy Assessment. Ophthalmologica. 2021;244(3): 250-257. | Internal validation only |
| Son J, Shin JY, Kim HD, Jung KH, Park KH, Park SJ. Development and Validation of Deep Learning Models for Screening Multiple Abnormal Findings in Retinal Fundus Images. Ophthalmology. 2020;127(1):85-94. | Outcomes |
| Sosale AR. Screening for diabetic retinopathy-is the use of artificial intelligence and cost-effective fundus imaging the answer? International Journal of Diabetes in Developing Countries. 2019;39(1). | Not a DTA study |
| Srivastava R, Wong DW, Lixin D, Jiang L, Tien Yin W. Red lesion detection in retinal fundus images using Frangi-based filters. Conf Proc IEEE Eng Med Biol Soc. 2015;2015:5663-6. | Internal validation only |
| Stevenson CH, Hong SC, Ogbuehi KC. Development of an artificial intelligence system to classify pathology and clinical features on retinal fundus images. Clin Experiment Ophthalmol. 2019;47(4):484-9. | Internal validation only |
| Stolte S, Fang R. A survey on medical image analysis in diabetic retinopathy. Med Image Anal. 2020;64:101742. | Not a DTA study |
| Sumathy B, Poornachandra S. Automated dr and prediction of various related diseases of retinal fundus images. Biomedical Research (India). 2018;2018(Special Issue ArtificialIntelligentTechniquesforBioMedicalSignalProcessingEdition-II):S325-S32. | Internal validation only |
| Tang HL, Goh J, Peto T, Ling BW, Al Turk LI, Hu Y, et al. The reading of components of diabetic retinopathy: an evolutionary approach for filtering normal digital fundus imaging in screening and population based studies. PLoS ONE. 2013;8(7):e66730. | Internal validation only |
| Thomas SA, Titus G. Design of a portable retinal imaging module with automatic abnormality detection. Biomedical Signal Processing and Control. 2020;60 (no pagination)(101962). | Internal validation only |
| Ting DSJ, Foo VH, Yang LWY, Sia JT, Ang M, Lin H, et al. Artificial intelligence for anterior segment diseases: Emerging applications in ophthalmology. British Journal of Ophthalmology. 2020;12:12. | Not a DTA study |
| Ting DSW, Bellemo V, Hamzah H, Tan GSW, Wong TY, Wong TH et al. Artificial intelligence-assisted diabetic retinopathy screening program: A 5-year bench to bedside translational study. Investigative Ophthalmology and Visual Science. 2020;61(7). | Not a full report |
| Ting DSW, Carin L, Abramoff MD. Observations and Lessons Learned From the Artificial Intelligence Studies for Diabetic Retinopathy Screening. JAMA Ophthalmology. 2019;13:13. | Not a DTA study |
| Ting DSW, Cheung CY, Nguyen Q, Sabanayagam C, Lim G, Lim ZW, et al. Deep learning in estimating prevalence and systemic risk factors for diabetic retinopathy: a multi-ethnic study. npj digit. 2019;2:24. | Aim |
| Ting DSW, Pasquale LR, Peng L, Campbell JP, Lee AY, Raman R, et al. Artificial intelligence and deep learning in ophthalmology. British Journal of Ophthalmology. 2019;103(2):167. | Not a DTA study |
| Ting DSW, Peng L, Varadarajan AV, Keane PA, Burlina PM, Chiang MF, et al. Deep learning in ophthalmology: The technical and clinical considerations. Prog Retin Eye Res. 2019;72:100759. | Not a DTA study |
| Tobin KW, Abramoff MD, Chaum E, Giancardo L, Govindasamy V, Karnowski TP, et al. Using a patient image archive to diagnose retinopathy. Conf Proc IEEE Eng Med Biol Soc. 2008;2008:5441-4. | Internal validation only |
| Tobin KW, Chaum E, Govindasamy VP, Karnowski TP. Detection of anatomic structures in human retinal imagery. IEEE Trans Med Imaging. 2007;26(12):1729-39. | Internal validation only |
| Torok Z, Peto T, Csosz E, Tukacs E, Molnar AM, Berta A, et al. Combined Methods for Diabetic Retinopathy Screening, Using Retina Photographs and Tear Fluid Proteomics Biomarkers. J Diabetes Res. 2015;2015:623619. | Internal validation only |
| Tsai CL, Madore B, Leotta MJ, Sofka M, Yang G, Majerovics A, et al. Automated retinal image analysis over the internet. IEEE Trans Inf Technol Biomed. 2008;12(4):480-7. | Aim |
| Ullah H, Saba T, Islam N, Abbas N, Rehman A, Mehmood Z, et al. An ensemble classification of exudates in color fundus images using an evolutionary algorithm based optimal features selection. Microsc Res Tech. 2019;82(4):361-72. | Internal validation only |
| Umadevi KS, Jeyapriya J. Cascaded neural network based automated detection of diabetic retinopathy. Indian Journal of Public Health Research and Development. 2017;8(4):1322-8. | Internal validation only |
| Usher D, Dumskyj M, Himaga M, Williamson TH, Nussey S, Boyce J. Automated detection of diabetic retinopathy in digital retinal images: a tool for diabetic retinopathy screening. Diabetic Medicine. 2004;21(1):84-90. | Internal validation only |
| Valverde C, Garcia M, Hornero R, Lopez-Galvez MI. Automated detection of diabetic retinopathy in retinal images. Indian J Ophthalmol. 2016;64(1):26-32. | Not a DTA study |
| van Grinsven MJ, van Ginneken B, Hoyng CB, Theelen T, Sanchez CI. Fast Convolutional Neural Network Training Using Selective Data Sampling: Application to Hemorrhage Detection in Color Fundus Images. IEEE Trans Med Imaging. 2016;35(5):1273-84. | Outcomes |
| Venkatesan R, Chandakkar P, Li B, Li HK. Classification of diabetic retinopathy images using multi-class multiple-instance learning based on color correlogram features. Conf Proc IEEE Eng Med Biol Soc. 2012;2012:1462-5. | Internal validation only |
| Verbraak FD, Schmidt-Erfurth U, Grzybowski A, Abramoff M, Schlingemann R. Is automated screening for diabetic retinopathy indeed not yet ready as stated by Grauslund et al.? Acta Ophthalmologica. 2020;98(2):e257-e8. | Not a DTA study |
| Vidal-Alaball J, Royo Fibla D, Zapata MA, Marin-Gomez FX, Solans Fernandez O. Artificial Intelligence for the Detection of Diabetic Retinopathy in Primary Care: Protocol for Algorithm Development. JMIR Res Protoc. 2019;8(2):e12539. | Not a DTA study |
| Vijayabaskar J, Rajeswari D, Vaithiyanathan V. To detect diabetic retinopathy in fundus enhanced retina images using effective ROI segmentation and kirch's templates. International Journal of Pharmacy and Technology. 2016;8(4):23240-52. | Internal validation only |
| Vijayalakshmi R, Selvarajan S. A decision support system for detecting the stages of diabetic retinopathy by using fundus images. Journal of Pure and Applied Microbiology. 2015;9(Special Edition):65-70. | Full text not found |
| Vollmer S, Mateen BA, Bohner G, Kiraly FJ, Ghani R, Jonsson P, et al. Machine learning and artificial intelligence research for patient benefit: 20 critical questions on transparency, replicability, ethics, and effectiveness. The BMJ. 2020;368 (no pagination)(l6927). | Not a DTA study |
| Wang H, Yuan G, Zhao X, Peng L, Wang Z, He Y, et al. Hard exudate detection based on deep model learned information and multi-feature joint representation for diabetic retinopathy screening. Comput Methods Programs Biomed. 2020;191:105398. | Outcomes |
| Wang J, Bai Y , Xia B. Simultaneous Diagnosis of Severity and Features of Diabetic Retinopathy in Fundus Photography Using Deep Learning. IEEE journal of biomedical and health informatics 24(12): 3397-3407 | Internal validation only |
| Wang R, Chen B, Meng D, Wang L. Weakly Supervised Lesion Detection From Fundus Images. IEEE Trans Med Imaging. 2019;38(6):1501-12. | Internal validation only |
| Wang S, Summers RM. Machine learning and radiology. Med Image Anal. 2012;16(5):933-51. | Not a DTA study |
| Wang S, Tang HL, Al Turk LI, Hu Y, Sanei S, Saleh GM, et al. Localizing Microaneurysms in Fundus Images Through Singular Spectrum Analysis. IEEE Trans Biomed Eng. 2017;64(5):990-1002. | Outcomes |
| Wang S, Yin Y, Cao G, Wei B, Zheng Y, Yang G. Hierarchical retinal blood vessel segmentation based on feature and ensemble learning. Neurocomputing. 2015;149(PB):708-17. | Outcomes |
| Wang S, Zhang Y, Lei S, Zhu H, Li J, Wang Q, et al. Performance of deep neural networkbased artificial intelligence method in diabetic retinopathy screening: a systematic review and meta-analysis of diagnostic test accuracy. Eur. 2020;183(1):41-9 | Not a DTA study |
| Wang XN, Dai L, Li ST, Kong HY, Sheng B, Wu Q. Automatic Grading System for Diabetic Retinopathy Diagnosis Using Deep Learning Artificial Intelligence Software. Curr Eye Res. 2020:1-6. | Poorly written paper |
| Waymel Q, Badr S, Demondion X, Cotten A, Jacques T. Impact of the rise of artificial intelligence in radiology: What do radiologists think? Diagnostic and Interventional Imaging. 2019;100(6):327-36. | Not a DTA study |
| Welikala RA, Dehmeshki J, Hoppe A, Tah V, Mann S, Williamson TH, et al. Automated detection of proliferative diabetic retinopathy using a modified line operator and dual classification. Comput Methods Programs Biomed. 2014;114(3):247-61. | Internal validation only |
| Welikala RA, Fraz MM, Dehmeshki J, Hoppe A, Tah V, Mann S, et al. Genetic algorithm based feature selection combined with dual classification for the automated detection of proliferative diabetic retinopathy. Comput Med Imaging Graph. 2015;43:64-77. | Internal validation only |
| Wong TY, Sabanayagam C. Strategies to Tackle the Global Burden of Diabetic Retinopathy: From Epidemiology to Artificial Intelligence. Ophthalmologica. 2020;243(1):9-20. | Not a DTA study |
| Wong TY, Sabanayagam C. The War on Diabetic Retinopathy: Where Are We Now? Asia Pac J Ophthalmol (Phila). 2019;8(6):448-56. | Not a DTA study |
| Wong TY. Artificial intelligence in ophthalmology: Concepts, progress, challenges and myths synopsis. Clinical and Experimental Ophthalmology. 2019;47 (Supplement 1):15-6. | Not a DTA study |
| Xiang Y, Zhao L, Liu Z, Wu X, Chen J, Long E, et al. Implementation of artificial intelligence in medicine: Status analysis and development suggestions. Artificial Intelligence in Medicine. 2020;102:101780. | Not a DTA study |
| Xie Y, Nguyen QD, Hamzah H, Lim G, Bellemo V, Gunasekeran DV, et al. Artificial intelligence for teleophthalmology-based diabetic retinopathy screening in a national programme: an economic analysis modelling study. The Lancet Digital Health. 2020;2(5):e240-e9. | Not a DTA study |
| Xu K, Feng D, Mi H. Deep Convolutional Neural Network-Based Early Automated Detection of Diabetic Retinopathy Using Fundus Image. Molecules (Basel). 2017;22(12):23. | Internal validation only |
| Xu L, Luo S. Optimal algorithm for automatic detection of microaneurysms based on receiver operating characteristic curve. J Biomed Opt. 2010;15(6):065004. | Internal validation only |
| Yao L, Zhong Y, Wu J, Zhang G, Chen L, Guan P, et al. Multivariable Logistic Regression And Back Propagation Artificial Neural Network To Predict Diabetic Retinopathy. Diabetes Metab Syndr Obes. 2019;12:1943-51. | Aim |
| Yedururi S, Katabathina VS, Jo NH, Rachamallu M, Prasad S, Marcal L. Machine learning and artificial intelligence in oncologic imaging: Potential barriers and solutions, abdominal imagers' perspective. Cancer Imaging Conference: 19th Meeting and Annual of the International Cancer Imaging Society Italy. 2019;19(Supplement 1). | Not a DTA study |
| Yip MYT, Lim G, Lim ZW, Nguyen QD, Chong CCY, Yu M, et al. Technical and imaging factors influencing performance of deep learning systems for diabetic retinopathy. npj digit. 2020;3:40. | Aim |
| You Z, Hu X, Shi K. Will artificial intelligence replace ophthalmologist in diabetic retinopathy screening? Biomedical Research (India). 2017;28(15):6920. | Not a DTA study |
| Yun WL, Mookiah MRK, Koh JEW. Automated detection of proliferative diabetic retinopathy using brownian motion features. Journal of Medical Imaging and Health Informatics. 2014;4(2):250-4. | Internal validation only |
| Zago GT, Andreao RV, Dorizzi B, Teatini Salles EO. Diabetic retinopathy detection using red lesion localization and convolutional neural networks. Computers in biology and medicine. 2020;116: 103537. | Internal validation only |
| Zaki WMDW, Zulkifley MA, Hussain A, Halim WHWA, Mustafa NBA, Ting LS. Diabetic retinopathy assessment: Towards an automated system. Biomedical Signal Processing and Control. 2016;24:72-82. | Not a DTA study |
| Zapata MA, Royo-Fibla D, Font O, Vela JI, Marcantonio I, Moya-Sanchez EU, et al. Artificial Intelligence to Identify Retinal Fundus Images, Quality Validation, Laterality Evaluation, Macular Degeneration, and Suspected Glaucoma. Clinical Ophthalmology. 2020;14:419-29. | Target condition |
| Zhang L, Feng S, Duan G, Li Y, Liu G. Detection of Microaneurysms in Fundus Images Based on an Attention Mechanism. Genes (Basel). 2019;10(10):17. | Internal validation only |
| Zheng R, Liu L, Zhang S, Zheng C, Bunyak F, Xu R, et al. Detection of exudates in fundus photographs with imbalanced learning using conditional generative adversarial network. Biomedical Optics Express. 2018;9(10):4863-78. | Outcomes |
| Zhou K, Gu Z, Liu W, Luo W, Cheng J, Gao S, et al. Multi-Cell Multi-Task Convolutional Neural Networks for Diabetic Retinopathy Grading. Conf Proc IEEE Eng Med Biol Soc. 2018;2018:2724-7. | Internal validation only |
| Zhou W, Wu C, Chen D, Wang Z, Yi Y, Du W. Automated Detection of Red Lesions Using Superpixel Multichannel Multifeature. Comput. 2017;2017:9854825. | Internal validation only |
| Zutis K, Trucco E, Hubschman JP, Reed D, Shah S, van Hemert J. Towards automatic detection of abnormal retinal capillaries in ultra-wide-field-of-view retinal angiographic exams. Conf Proc IEEE Eng Med Biol Soc. 2013;2013:7372-5. | Target condition |

**Table S5 Included ARIASs and number of studies evaluating each system**

| **ARIAS (Company/Institution, Country)** | **DL/ML** | **N of studies** |
| --- | --- | --- |
| Airdoc (Airdoc, Beijing, China) | DL | 1 |
| Deep Learning Platform, DLP (multiple institutions, China) | DL | 1 |
| DAPHNE (University of Surrey, UK) | DL | 1 |
| DART (TeleDx, Santiago, Chile) | DL | 1 |
| DeepDR (multiple institutions, Shanghai, China)^1^ | DL | 1 |
| Deep Learning Algorithm, DLA (Universitat Rovira & Virgili, Spain) | DL | 2 |
| EyeArt (Eyenuk Inc, Woodland Hills, CA, USA) | DL  ML | 6  2 |
| EyeGrader (Guangzhou Healgoo Interactive Medical Technology Co, China) | DL | 2 |
| EyeWisdon (n/a, China) | DL | 1 |
| Google (Google LLC, Mountain View, California, USA) | DL | 4 |
| IDx-DR (Digital Diagnostics, Coralville, IA, USA) | DL | 6 |
| iGradingM (Medalytix, Liverpool, UK) and the Aberdeen system (University of Aberdeen, UK) | ML | 6 |
| Kanagasingam 2018 (multiple institutions, Australia and USA) | DL | 1 |
| RetCAD (Thritona, Nijmegen, the Netherlands) | DL | 1 |
| RetinaLyze (RetinaLyze System A/S, Copenhagen, Denmark) | ML | 3 |
| RetmarkerSR (Retmarker SA, Meteda, Coimbra, Portugal) | ML | 4 |
| SELENA™ (National Health Innovation Centre, Singapore) | DL | 2 |
| Visiona (Visiona Medtech International Ltd, Hong Kong, China) | DL | 1 |
| ^1^One more study evaluating DeepDR, Wang 2020^49^, was excluded due to poor presentation (see the footnotes in Table 2 for more details)  Two studies evaluated more than one ARIAS |  |  |

**Table S6 For each AI-system, the table shows study-level sensitivity, specificity, PPV and NPV arranged by prevalence in ascending order**

| Test | Study | Prevalence | Sensitivity | Specificity | PPV | NPV |
| --- | --- | --- | --- | --- | --- | --- |
| Airdoc | He 2020 | 11% | 91% | 99% | 92% | 99% |
| Cen 2021 | Cen 2021 | 46% | 98% | 100% | 100% | 98% |
| DAPHNE | Al Turk 2020 | 12% | 96% | 91% | 58% | 99% |
| DART | Arenas-Cavalli 2021 | 19% | 94% | 73% | 45% | 98% |
| DeepDR | Dai 2021 | 13% | 94% | 88% | 54% | 99% |
| DLA | Romero-Aroca 2020 | 7% | 100% | 97% | 70% | 100% |
| DLA | Baget-Bernaldiz 2021 | 11% | 96% | 100% | 99% | 100% |
| EyeArt v1 | Tufail 2016 | 14% | 94% | 16% | 15% | 94% |
| EyeArt v1 | Bhaskaranand 2016 | 17% | 90% | 63% | 33% | 97% |
| EyeArt_v2 | Heydon 2020 | 7% | 96% | 54% | 14% | 99% |
| EyeArt_v2 | Olivera-Barrios 2020 | 9% | 96% | 56% | 17% | 99% |
| EyeArt_v2 | FDA 2020 | 16% | 93% | 86% | 54% | 98% |
| EyeArt_v2 | Bhaskaranand 2019 | 20% | 91% | 91% | 72% | 98% |
| EyeArt_v2 | Liu 2020 | 26% | 100% | 66% | 50% | 100% |
| EyeArt_v2 | Sarao 2020 | 47% | 91% | 75% | 77% | 90% |
| EyeGrader | Li 2018 | 3% | 93% | 98% | 65% | 100% |
| EyeGrader | Keel 2018 | 14% | 92% | 94% | 71% | 99% |
| EyeWisdom | Ming 2021 | 5% | 85% | 98% | 69% | 99% |
| Google | Krause 2018 | 12% | 97% | 92% | 63% | 100% |
| Google | Raumviboonsuk 2019 | 12% | 97% | 96% | 75% | 100% |
| Google | Gulshan 2016 | 20% | 91% | 94% | 79% | 98% |
| Google | Gulshan 2019 | 31% | 92% | 95% | 89% | 96% |
| IDx-DR | van der Heijden 2018 | 2% | 91% | 84% | 13% | 100% |
| IDx-DR | Shah 2020 | 4% | 100% | 82% | 19% | 100% |
| IDx-DR | Verbraak 2019 | 5% | 79% | 94% | 39% | 99% |
| IDx-DR | Abramoff 2016 | 21% | 97% | 87% | 67% | 99% |
| IDx-DR | Abramoff 2018 | 24% | 87% | 90% | 73% | 96% |
| iGrading | Fleming 2010a | 7% | 98% | 41% | 11% | 100% |
| iGrading | Goatman 2011 | 7% | 99% | 70% | 21% | 100% |
| iGrading | Soto-Pedre 2015 | 16% | 95% | 69% | 36% | 99% |
| iGrading | Fleming 2010b | 18% | 95% | 51% | 30% | 98% |
| iGrading | Philip 2007 | 38% | 90% | 67% | 63% | 92% |
| Kanagasingam 2018 | Kanagasingam 2018 | 1% | 100% | 92% | 12% | 100% |
| RedCAD | Gonzalez-Gonzalo 2020 | 42% | 92% | 92% | 89% | 94% |
| RetinaLyze | Bouhaimed 2008 | 18% | 88% | 51% | 28% | 95% |
| RetmarkerSR | Figueiredo 2015 | 9% | 89% | 59% | 17% | 98% |
| RetmarkerSR | Oliveira 2011 | 9% | 96% | 52% | 16% | 99% |
| RetmarkerSR | Tufail 2016 | 14% | 85% | 48% | 21% | 95% |
| SELENA | Ting 2017 | 3% | 90% | 92% | 25% | 100% |
| SELENA | Bellemo 2019 | 23% | 92% | 89% | 71% | 98% |
| Visiona | Ramachandran 2018 | 3% | 85% | 80% | 10% | 99% |
| PPV – positive predictive value, NPV – negative predictive value  Hansen 2004 (RetinaLyze) and Grzybowski 2021 (RetinaLyze and IDx-DR) are excluded as they used two-gate (case controlled) design. There are some minor discrepancies between the results reported here and Table 3, which presents the accuracy outcomes as reported in the papers. This is either because the study reported multiple pairs of sensitivity and specificity (e.g. mydriasis vs no mydriasis) and we had to select a single set, or because the 2x2 data had to be recalculated from the reported sensitivities and specificities. | | | | | | |

**Table S7 The table shows study-level sensitivity, specificity, PPV and NPV arranged by prevalence and grouped by 10% increments**

| Test | Study | Prevalence | Sensitivity | Specificity | PPV | NPV |
| --- | --- | --- | --- | --- | --- | --- |
| Kanagasingam 2018 | Kanagasingam 2018 | 1% | 100% | 92% | 77% | 90% |
| IDx-DR | van der Heijden 2018 | 2% | 91% | 84% | 100% | 98% |
| Visiona | Ramachandran 2018 | 3% | 85% | 80% | 89% | 94% |
| EyeGrader | Li 2018 | 3% | 93% | 98% | 63% | 92% |
| SELENA | Ting 2017 | 3% | 90% | 92% | 89% | 96% |
| IDx-DR | Shah 2020 | 4% | 100% | 82% | 50% | 100% |
| EyeWisdom | Ming 2021 | 5% | 85% | 98% | 73% | 96% |
| IDx-DR | Verbraak 2019 | 5% | 79% | 94% | 71% | 98% |
| iGrading | Fleming 2010a | 7% | 98% | 41% | 67% | 99% |
| DLA | Romero-Aroca 2020 | 7% | 100% | 97% | 72% | 98% |
| EyeArt_v2 | Heydon 2020 | 7% | 96% | 54% | 79% | 98% |
| iGrading | Goatman 2011 | 7% | 99% | 70% | 45% | 98% |
| RetmarkerSR | Figueiredo 2015 | 9% | 89% | 59% | 30% | 98% |
| RetmarkerSR | Oliveira 2011 | 9% | 96% | 52% | 28% | 95% |
| EyeArt_v2 | Olivera-Barrios 2020 | 9% | 96% | 56% | 33% | 97% |
| DLA | Baget-Bernaldiz 2021 | 11% | 96% | 100% | 54% | 98% |
| Airdoc | He 2020 | 11% | 91% | 99% | 36% | 99% |
| DAPHNE | Al Turk 2020 | 12% | 96% | 91% | 15% | 94% |
| Google | Krause 2018 | 12% | 97% | 92% | 21% | 95% |
| Google | Raumviboonsuk 2019 | 12% | 97% | 96% | 71% | 99% |
| DeepDR | Dai 2021 | 13% | 94% | 88% | 54% | 99% |
| EyeGrader | Keel 2018 | 14% | 92% | 94% | 75% | 100% |
| EyeArt v1 | Tufail 2016 | 14% | 94% | 16% | 63% | 100% |
| RetmarkerSR | Tufail 2016 | 14% | 85% | 48% | 58% | 99% |
| iGrading | Soto-Pedre 2015 | 16% | 95% | 69% | 92% | 99% |
| EyeArt_v2 | FDA 2020 | 16% | 93% | 86% | 99% | 100% |
| EyeArt v1 | Bhaskaranand 2016 | 17% | 90% | 63% | 17% | 99% |
| RetinaLyze | Bouhaimed 2008 | 18% | 88% | 51% | 16% | 99% |
| iGrading | Fleming 2010b | 18% | 95% | 51% | 17% | 98% |
| DART | Arenas-Cavalli 2021 | 19% | 94% | 73% | 21% | 100% |
| Google | Gulshan 2016 | 20% | 91% | 94% | 14% | 99% |
| EyeArt_v2 | Bhaskaranand 2019 | 20% | 91% | 91% | 70% | 100% |
| IDx-DR | Abramoff 2016 | 21% | 97% | 87% | 11% | 100% |
| SELENA | Bellemo 2019 | 23% | 92% | 89% | 39% | 99% |
| IDx-DR | Abramoff 2018 | 24% | 87% | 90% | 69% | 99% |
| EyeArt_v2 | Liu 2020 | 26% | 100% | 66% | 19% | 100% |
| Google | Gulshan 2019 | 31% | 92% | 95% | 25% | 100% |
| iGrading | Philip 2007 | 38% | 90% | 67% | 65% | 100% |
| RedCAD | Gonzalez-Gonzalo 2020 | 42% | 92% | 92% | 10% | 99% |
| Cen 2021 | Cen 2021 | 46% | 98% | 100% | 13% | 100% |
| EyeArt_v2 | Sarao 2020 | 47% | 91% | 75% | 12% | 100% |
| PPV – positive predictive value, NPV – negative predictive value  Hansen 2004 (RetinaLyze) and Grzybowski 2021 (RetinaLyze and IDx-DR) are excluded as they used two-gate (case controlled) design. There are some minor discrepancies between the results reported here and Table 3, which presents the accuracy outcomes as reported in the papers. This is either because the study reported multiple pairs of sensitivity and specificity (e.g. mydriasis vs no mydriasis) and we had to select a single set, or because the 2x2 data had to be recalculated from the reported sensitivities and specificities. | | | | | | |

**Table S8 Test accuracy at other thresholds and factors affecting accuracy**

| **Study** | **Any DR, % (95% CI)** | **Higher grades DR, % (95% CI)** | **Ungradable by ARIAS** | **Factors affecting performance** |
| --- | --- | --- | --- | --- |
| **Airdoc (DL)** |  |  |  |  |
| He 2020 | SE 90.79 (86.4 to 94.1)  SP 98.5 (97.8 to 99.0) | PDR: SE 80.36 (67.6 to 89.8)  SP 99.42 (98.9 to 99.7) | None reported | N/A |
| **DAPHNE (DL)** |  |  |  |  |
| Al Turk 2020 | N/A | PDR vs Non-PDR: SE 97.18% (91.2% to 99.6%); SP 87.77% (85.3% to 88.80%) | 5% across the 3 datasets (China, Kenya and Saudi Arabia) | N/A |
| **DART (DL)** |  |  |  |  |
| Arenas-Cavalli 2021 | Including ungradable cases:  SE 94.6 (90.9 to 96.9)  SP 74.3 (73.3 to 75.0)  Excluding ungradable cases:  SE 96.3, SP 74.3 | FN rate (complement of SE):  3.65% for ≥ R1 or DMO  3.54% for ≥R2 or DMO  4.27% for ≥R3 or DMO  5.38% for R4 or DMO | 1.8% (20 out of 1123) | N/A |
| **DeepDR (DL)** |  |  |  |  |
| Dai 2021 | SE 87.6 (87.4 to 87.8)  SP 83.1 (82.5 to 83.7) | Moderate NPDR:  SE 89.0 (88.4 to 89.6)  SP 79.3 (79.0 to 79.6)  Severe NPDR:  SE 91.8 (90.5 to 92.9)  SP 88.0 (87.8 to 88.2)  PDR:  SE 92.7 (90.0 to 95.0)  SP 85.5 (85.2 to 85.7) | N/A (but in the local validation dataset it achieved AUC: 0.938 for identification of artefacts, 0.920 for clarity and 0.968 for field definition | N/A |
| **DLA (DL)** |  |  |  |  |
| Baget-Bernaldiz 2021 | SE 97.92 (97.26 to 98.46)  SP 99.91 (99.83 to 99.95) | N/A (but see table 2 above for distribution of FNs) | 0.98% | N/A |
| Romero-Aroca 2020 | SE 96.7, SP 97.6 | Unclear | 0.69% (another 0.22% classified as ungradable by the RS but missed by the DL) | N/A |
| **EyeArt ML&DL** |  |  |  |  |
| Bhaskaranand 2016 (ML) | N/A | STDR: SE 96.8 | 1.4% | N/A |
| Bhaskaranand 2019 | N/A | Of 5446 encounters with more than moderate NPDR and/or DMO, 5363 were classified as ‘refer’, SE 98.5 | 0.9%, treated as ‘refer’ | Non-mydriasis vs mydriasis:  SE 89.6% vs 93.0%  SP 91.7% vs 90.4%  Treatable DR: SE 98.0% vs 98.8%  Non-screenable: 1.2% vs 0.6% |
| FDA 2020 | N/A | Images ungradable by ARIAS are included in the SE & SP estimates  VTDR in the sequentially enrolled cohort:  Primary care (n=45 subjects):  SE 100 (51.0 to 100)  SP 97.5 (93.4 to 100)  Imageability 96.5 (90.6 to 100)  Ophthalmology (n=190 subjects):  SE 88.9 (no CI)  SP 93.8 (90.4 to 96.6)  Imageability 98.6 (97.0 to 99.7)  VTDR in the enrichment permitted cohort:  Primary care (n=335 subjects):  SE 91.7 (80.0 to 100)  SP 92.2 (89.6 to 94.6)  Imageability 96.7 (94.8 to 98.5)  Ophthalmology (n=85 subjects):  SE 100 (51.0 to 100)  SP 89.8 (83.9 to 95.4)  Imageability 97.0 (92.9 to 100) | Imageability ≥96.5% across cohorts | In the sequential enrolment cohort dilation improved imageability from 94.2% to 96.5% of eyes (primary care) and from 81.7% to 98.6% (ophthalmology); and, in the enrichment-permitted cohort from 89.0% to 96.7% (primary care) and from 83.5% to 96.5% (ophthalmology) |
| Heydon 2020 | Detection rate for:  R1M0 89.1 (88.4 to 89.9) | Detection rate:  R1M1: 98.3 (97.3 - 98.9)  R2: 100 (98.7 - 100)  R3: 100 (97.9 - 100) | Detection rate 89.4% (87.0% - 91.5%) | In R1M0 and R1M1: older individuals more likely to test positive; small effect of ethnicity and no effect of sex (personal communication) |
| Liu 2020 | N/A | VTDR: SE 100 | 29.4% inconclusive results | N/A |
| Olvera-Barrios 2020 | SE 92.26 (88.37 to 94.69)  SP 74 (no CI) | VTDR: SE 100 (99.53 to 100)  PDR: SE 100 (61 to 100) | N/A (but classified as ‘disease absent’ 30.77% of the images classified by the RS as ungradable) | Similar performance when used with true- colour, wide- field confocal scanning images (the EIDON platform, CenterVue, Padua, Italy) |
| Sarao 2020 | N/A | N/A | 1.2% (conventional flush fundus camera) | Camera type, conventional flash fundus vs white LED: difference between AUC (95%CI, p value) 0.0737 (95% CI 0.0263–0.121), p =0.0023 |
| Tufail 2016 (ML) | SE 94.7 (94.2 to 95.2)  SP 20 (19 to 21) | Detection rate at RDR threshold:  R2 (M0 & M1) 99.4% (98.3% to 99.8%); R3 (M0 & M1) 99.6% (97.0% to 99.9%) | Treated as ‘refer’; the software classified 23% of manually ungradable as ‘no refer’ | SE and FPR were not affected by ethnicity, sex or camera type, but SE was marginally lower with increasing patient age |
| **EyeGrader (DL)** |  |  |  |  |
| Keel 2018 | N/A | N/A | 3.1% of participants had ungradable photographs in both eyes, 10.4% gradable in one eye only | N/A |
| Li 2018 | N/A | N/A | 1.9% | N/A |
| **EyeWisdom (DL)** | | | | |
| Ming 2021 | SE 90.0 (68.3 to 98.8),  SP 96.6 (92.1 to 98.9) | N/A | 41/386 eyes were classified as ‘ungradable’ by the system, of which 15 had no DR and 26 were ungradable by the RS; the RS found additional 24 ‘ungradable’ eyes, of which 2 were graded as moderate NPDR and 22 as no DR by the system | N/A |
| **Google AI (DL)** |  |  |  |  |
| Gulshan 2016 | N/A | High SP operating point Moderate or worse diabetic retinopathy:  SE 90.1 (87.2 to 92.6)  SP 98.2 (97.8 to 98.5)  Severe or worse DR only:  SE 84.0 (75.3 to 90.6)  SP 98.8 (98.5 to 99.0)  DMO only:  SE 90.8 (86.1 to 94.3)  SP 98.7 (98.4 to 99.0) | Image gradability:  SE 93.9 (93.3 to 94.5)  SP 90.0 (88.9 to 92.7) | Mydriatic vs non-mydriatic:  SE 89.6% (85.6% to 92.8%) vs 90.9% (86.1% to 94.4%)  SP 97.9% (97.3% to 98.4%) vs 98.5% (98.0% to 98.8%) |
| Gulshan 2019 | N/A | N/A | 110 images graded as ‘U’ by the adjudicators; of those 62 graded as referable after dilated fundus imaging; of the latter, 55 graded as ‘refer’ by ARIAS | N/A |
| Krause 2018 | SE 97.0, SP 91.7 | 100% detection rate for severe or PDR | None reported | Better performance when image resolution was 779x779 pixels relative to 299x299 pixels; Method of adjudication in the RS grading also had an impact (consensus better than majority decision) |
| Raumviboonsuk 2019 | N/A | Severe NPDR or worse: SE 96.7% (no CI),  PDR: SE 95.5% (no CI) | 11.2% of all images graded for DR and 15.3% of all images graded for DMO | N/A |
| **IDx-DR (DL)** |  |  |  |  |
| Abramoff 2016 | N/A | At RDR threshold:  SE VTDR 100% (96.1% to 100%)  SE DMO 100% (95.6% to 100%)  At VTDR threshold  SE 100.0% (96.1% to 100.0%)  SP 90.8% (88.5% to 92.7%) | 4% of the subjects had at least one image deemed to be of insufficient quality by the quality algorithm run outside the device | SE was not statistically different from that of the ML-based version of the software but specificity improved: DL version (95% CI 84.2% to 89.4%) vs ML version (95% CI 55.7% to 63.0%) |
| Abramoff 2018 | N/A | SE for VTDR:  97.4 (86.2 to 99.9) against 4W-D RS  92.2 (81.1 to 97.8) against ‘4W-D + OCT’ RS | 4% (after excluding the ungradable by the RS) | No significant effects on sensitivity for age, sex, race, ethnicity, HbA1C, lens status, or site. Same for specificity, except that there was increased specificity in subjects over 65 (p=0.030) |
| Grzybowski 2021 | N/A | N/A | N/A | N/A |
| Shah 2020 | N/A | VTDR:  SE 100 (95 to 100)  SP 94.64 (94 to 95) | 11.4%, excluded from analysis (probably after excluding images classified as ungradable by the RS) | Sex, age over 65, and duration of diabetes >10 years had no effect on sensitivity (p > 0.05/3); sex had no effect on specificity (p > 0.655), but the system had a higher specificity in subjects with a diabetes duration <10 y (86% vs 71% (p < 0.0001); and higher specificity in patients <65 y (89% vs 79%, p < 0.0001) |
| van der Heijden 2018 | N/A | VTDR: ICDR  SE 62 (32 to 85)  SP 95 (93 to 96)  VTDR: EURODIAB  SE 64 (36 to 86)  SP 95 (93 to 96) | 66.3% classified as gradable by the system (while the RS classified 80.4% as gradable; unclear what proportion of those overlapped but all ungradable images were excluded from the analysis) | Considerable difference between ICDR and EURODIAB when used for reference grading, mostly with respect of classifying mild cases |
| Verbraak 2019 | N/A | VTDR:  SE 100 (77.1 to 100)  SP 97.8 (96.8 to 98.5) | 9.3% of the images classified as gradable by the RS (all ungradable images excluded from the analysis) | N/A |
| **RedCAD** |  |  |  |  |
| Gonzalez-Gonzalo 2020 | N/A | N/A | N/A | N/A |
| **SELENA (DL)** |  |  |  |  |
| Bellemo 2019 | N/A | VTDR SE: 99.42 (99.15 to 99.68)  DMO SE: 97.19 (96.61 to 97.77) | 0.3% | Age <60 vs ≥60 years, AUC 0.982 vs 0. 965, p = 0.018; women vs men, AUC 0.977 vs 0.973, p = 0.512; HbA1c <8 vs ≥8, 0.966 vs 0.977, p = 0.252 |
| Ting 2017 | N/A | VTDR:  SE 100 (90.97 to 100)  SP 81.4 (80.57 to 82.22) | Unclear | ARIAS showed comparable performance in different subgroups of patients stratified by age, sex, and glycaemic control; SE ≥90% for RDR in the multi-ethnic cohorts |
| **Visiona (DL)** |  |  |  |  |
| Ramachandran 2018 | N/A | N/A | 103/485 eyes excluded due to patient non-attendance or poor quality, as determined by the RS | N/A |
| **Other DL ARIASs** |  |  |  |  |
| Cen 2021 | N/A | N/A | N/A | N/A (but reported that the presence of stains on dirty lens looking like hemorrhage spots were the main cause of FPs in the Messidor data) |
| Kanagasingam 2018 | N/A | N/A | N/A | N/A |
| **iGrading** |  |  |  |  |
| Fleming 2010a | N/A | Detection rate:  R2: 100% (193/193)  R3: 100% (324/324)  R4 (PDP): 100% (180/180)  M1: 99.2% (384/387)  M2: 97.3% (1099/1130) | Detection rate for ungradable (U) as defined by RS: 99.8% (1824/1827) | No statistically significant differences between Caucasians, Asians and Afro-Caribbean for non-referable retinopathy (Pearson χ^2^ test p=0.38) or for patients requiring referral to ophthalmology, 6 month recall or slit-lamp examination (Fisher exact test p=0.82). |
| Fleming 2010b | N/A | SE for M2+R3+R4:  MA: 95.0% (93.5% to 96.1%)  MA&EX&HM 96.9% (95.7% to 97.8%) | MA: SE for U by RS:  98.6% (97.4% to 99.3%)  MA, EX & HM: SE for U by RS:  SE 98.8% (97.6% to 99.4%) | Software detecting MA, EX and HM had better SE for RDR than MA alone: 94.9% (95% CI 93.5 to 96.0) to 96.6% (95.4 to 97.4); this did not affect SP (~50%) |
| Goatman 2011 | SE range 89.9 (88.7 to 90.9) to 95.8 (95.0 to 96.5) across the 4 strategies | SE for R2 (pre-PDR) and R3 (PDR) was 100% across the 4 strategies | SE for detecting ungradable images ranged from 97.4 (95.1 to 98.6) to 99.1% (97.5 to 99.7) | MA vs MA+BH+EX modes of the software had similar accuracy;  Using 1- or 2-fields also produced similar results |
| Philip 2007 | SE 90.5 (89.3 to 91.6)  SP 67.4 (66.0 to 68.8) | M1 97.4%, R2 100%, M2 97.2%, R3 100%, R4 100% | SE 99.5 (98.4 to 99.8)  SP 84.4 (83.5 to 85.3), i.e.  Of the 553 patients with ungradable images (per RS) the system misclassified 3 as gradable; and of the 6169 patients with gradable images (per RS) failed to grade 62 | N/A |
| Soto-Pedre 2015 | SE 94.52 (92.56 to 96.49)  SP 68.77 (67.18 to 70.36) | N/A but all 31 FN cases were classified as mild NPDR | 26.1% (vs 2.03% classified ungradable by the RS) | N/A |
| Tufail 2016 | N/A (failed evaluation) | N/A | N/A | N/A |
| **RetinaLyze (ML)** |  |  |  |  |
| Bouhaimed 2008 | N/A | N/A | 15/96 patients had at least one image of low quality; visual inspection confirmed the low quality of those images | Two settings reported: ‘red lesions only’ vs ‘red & bright lesions’: SE 82% vs 88%; SP 75% vs 52% |
| Grzybowski 2021 | N/A | N/A | 1 out of 4 images for 1 patient | N/A |
| Hansen 2004 | As in table 4 | SE 100.0 for moderate/severe NPDR and PDR | No mydriasis: 6/83 of which 5 classified as ungradable by the RS; Mydriasis: 2/83 (the RS classified all 83 as gradable) | Mydriasis vs no mydriasis:  Any DR: SE 97.0% & SP 75.0% vs SE 89.9% & SP 85.7%  Moderate NPDR or worse: SE 100% in both conditions |
| **RetmarkerSR (ML)** |  |  |  |  |
| Figueiredo 2015 | N/A | N/A | N/A | In 2 of the 4 datasets performance was lower due to the presence of low quality images: SE 100% & 100% vs 89.3% & 92%; SP 70.7% & 73% vs 57.6% & 58.9% |
| Oliveira 2011 | N/A | SE 100 for PDR (‘urgent referral) 0.3% of case with NPDR with maculopathy (‘referral as soon as possible’) were missed | N/A (images classified as ungradable by the RS were excluded from the study) | N/A |
| Ribeiro 2015 | N/A | SE 100% for PDR | N/A (3.5% of all eyes in the whole programme were non-classifiable) | N/A |
| Tufail 2016 | SE 73.0 (72.0 to 74.0)  SP 53 (52 to 54) | Detection rate at RDR threshold:  R2 (M0 & M1) 98.9% (97.7% to 99.5%); R3 (M0 & M1) 99.1% (96.6% to 99.8%) | Treated as ‘refer’; classified 33% of manually ungradable as ‘no refer’ | Accuracy varied according to the patient’s age, ethnicity and camera type |
| FDA 2020: ‘Imageability’ is defined as the proportion of results classified as ‘gradable’ by the software out of all results classified as ‘gradable’ by the reference standard.  CI – confidence interval, DMO – Diabetic Macular Oedema, DR – Diabetic Retinopathy, EURODIAB - EURODIAB IDDM Complications Study, FN – false negative, FPR – false positive rate, ICDR - International Clinical Diabetic Retinopathy, NPDR – Non-Proliferative Diabetic Retinopathy, PDR – Proliferative Diabetic Retinopathy, RDR – Referable Diabetic Retinopathy, RS – Reference Standard, SE – Sensitivity, SP – Specificity, TP – true positive result, U – ungradable | | | | |
